# Supplementary material for: Implementing a Multifaceted Intervention among Internal Medicine Residents with Audit and Educative Data Feedback Significantly Reduces Low-Value Care in Hospitalized Patients
Source: J Clin Med. 2022 Apr 26;11(9):2435. doi: 10.3390/jcm11092435 (PMC9104072; doi:10.3390/jcm11092435)
Supplement: Supplementary file 1 [file jcm-11-02435-s001.zip › Supplementary File S1/Vignette_smarterindicateur_BZD.pdf]

La Tour Medical Group

smarter medicine
Choosing Wisely Switzerland

Module E-learning

Don't use benzodiazepines or other sedative-hypnotics in older adults as first choice for insomnia, agitation or delirium and avoid prescription at discharge.

Version 1.0 30.8.2020

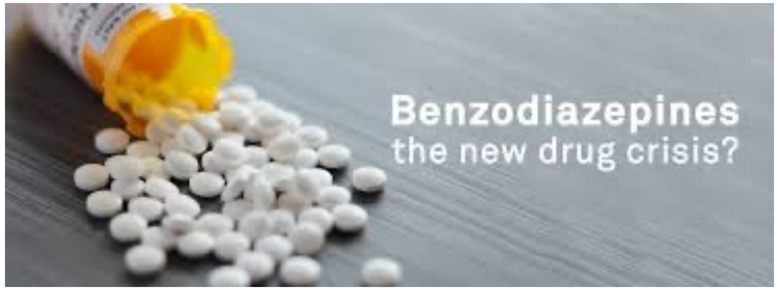

Commencer

Introduction

Large-scale studies consistently show that the **risk of car crashes**, **falls, and hip fractures** leading to hospitalization and death can **more than double** in older adults taking benzodiazepines and other sedative-hypnotics. **Older patients**, their caregivers, and their providers **should recognize these potential harms** when considering treatment strategies for insomnia, agitation, or delirium. **Use** of benzodiazepines should be **reserved for alcohol withdrawal symptoms**/delirium tremens or severe **generalized anxiety disorders** unresponsive to other therapies.

Sources: The American Geriatrics Society, The American Academy of Sleep Medicine, CH expert panel

Evidence level: Meta-analysis, Randomized Controlled Trials, Retrospective Cohort Studies, Guidelines

Continuer

Prescription rate

What proportion of patients admitted to the hospital are receiving new benzodiazepine treatment?

0-5% | 5-15% | 15-20% | 20-40%

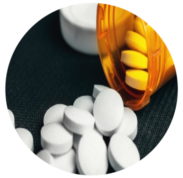

Place the drugs on the correct prescription rate

Valider

Benzodiazepines (BZDs) **are commonly prescribed** and are **often used**, on a long-term basis, **to treat** in the elderly population **sleep disorders and anxiety**[1].

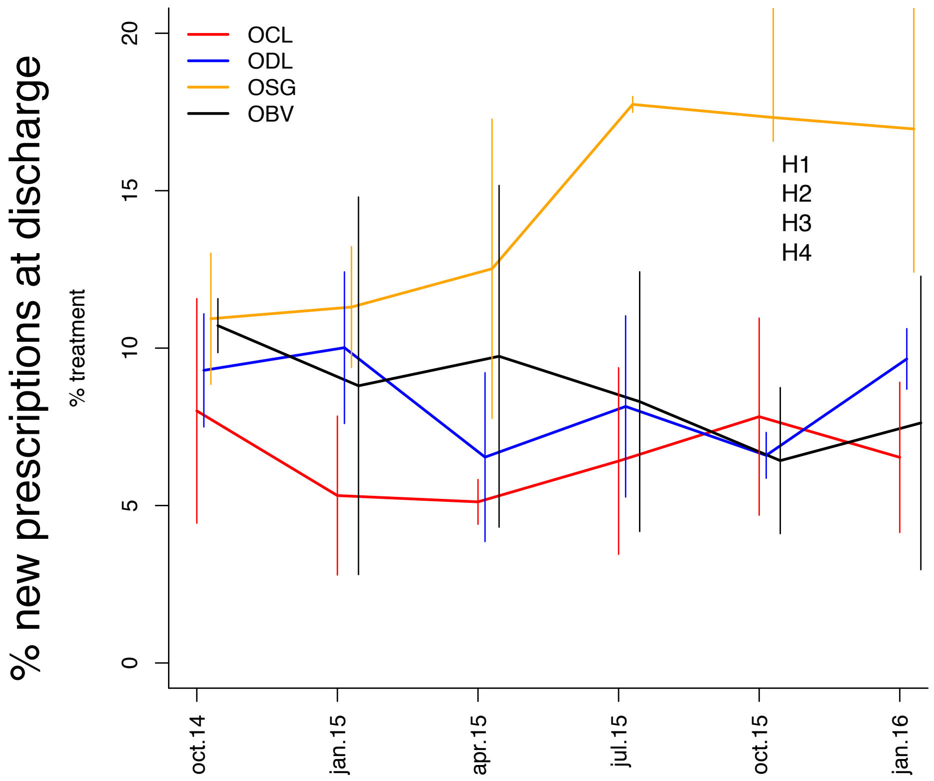

***Observational Study** on new BZDs prescriptions at discharge, **Internal Medicine** Services, 4 regional hospitals, N: 15'372, 18 Months (oct 2014 - march 2016)*
Prevalence of BZDs treatment at admission 35 ± 2 %

Swiss data

Sedative drugs were newly prescribed to 37% (n = 108) of patients during their stay.

*Among these, **37%** (n = 40) received a repeat prescription at discharge.*

1 Xing D et al., Osteoporos Int 2014; 25:105 –120

2 Schumacher et al., Drugs - Real World Outcomes 4, 225–234 (2017)

Complications

What is the most common complication in patients receiving benzodiazepine during their hospital stay?

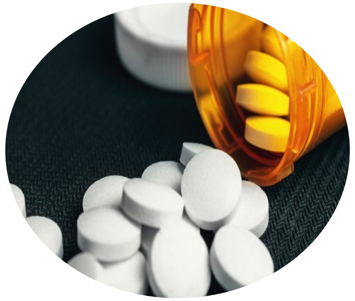

Addiction

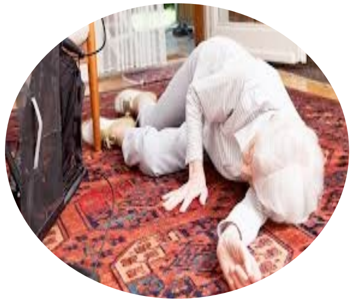

Fall and fracture

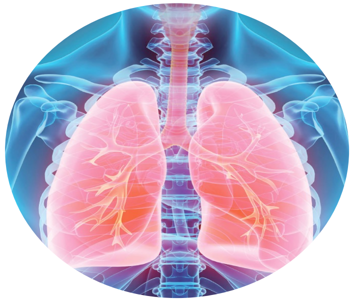

Respiratory depression

Choose the correct answer

Valider

BZD and risk for falls and fracture

- BZDs are considered as one of the **risk factors for falls and fractures** which lead, especially for the aging population, to **increased mortality and disability**[2].

- **29 %** of community-dwelling older adults **experience a fall each year** due to multiple factors (poor balance, effects of medication, sleep problems, etc)[3].

- The **cost of BZD-associated fall injuries in Europe** has recently been estimated as €1.5–2.2 billion per year[4].

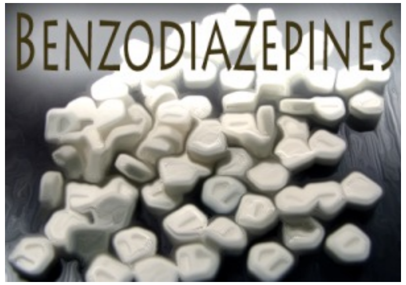

2 Allain H et al., Drugs Aging 2005; 22(9):749-65
3 Stone KL et al., Sleep Medicine 2008; 9 Suppl 1:S18-22
4 Panneman MJ et al., Drugs Aging 2003; 20(11):833-9

BZD use is associated with a significant increase in the risk of fractures in the elderly population

Adapted from Xing D et al., Association between use of benzodiazepines and risk of fractures: a **meta-analysis**, Osteoporos Int 2014

25 studies (19 case – control and 6 cohort studies)

Studies included	Relative risk		
	Number of studies	RR (95% CI)	P-value
All studies	25	1.25 (1.17-1.34)	< 0.001
Studies with participants aged ≥65 years	18	1.26 (1.15-1.38)	< 0.001
Studies that used hip fractures as outcome	17	1.35 (1.22-1.50)	< 0.001
Studies with methodological quality score ≥7	17	1.23 (1.15-1.31)	< 0.001

The relative fracture risk was increased by 26 % with participants aged ≥65 years.

[redacted]

During your night shift, you are called by nurse for a patient who complains of insomnia, the best answer is:

- A: Prescribe BZD (unique dose)
- B: Try non-drug treatment first
- C: Try a z compound drug (zolpidem)
- D: Pretend not answering

Are ‘Z’-compounds (i.e. zopiclone, zolpidem and zaleplon) a safer alternative to BZDs?

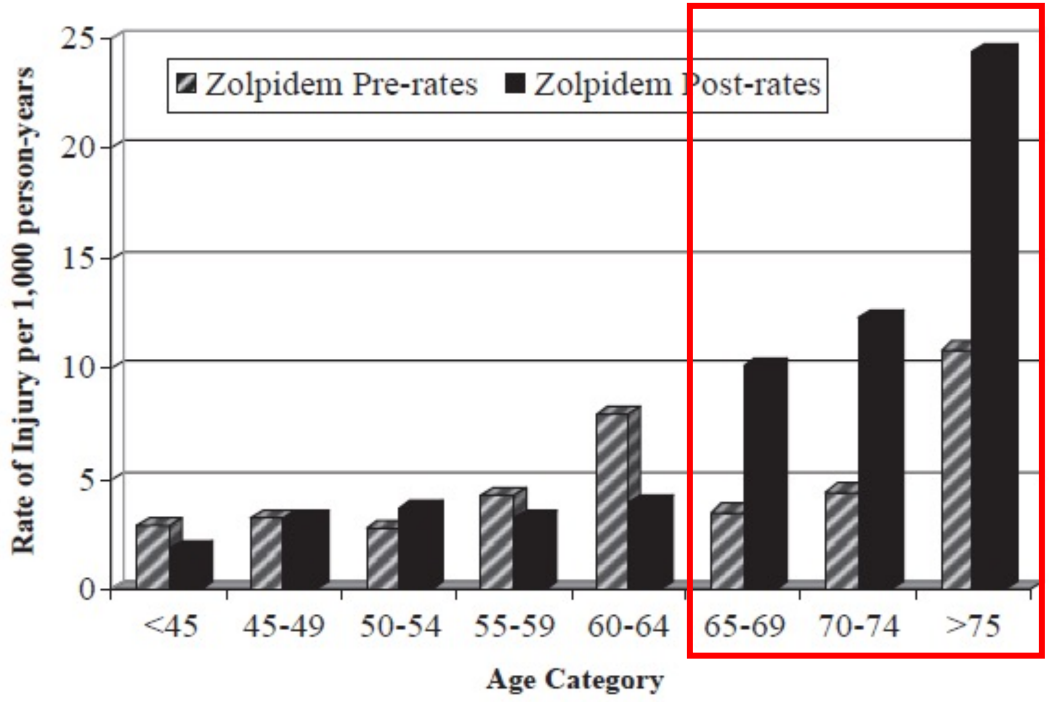

Finkle WD et al., Risk of Fractures Requiring Hospitalization After an Initial Prescription for Zolpidem, Alprazolam, Lorazepam, or Diazepam in Older Adults, J Am Geriatr 2011

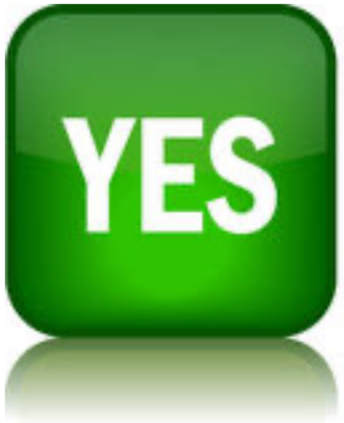

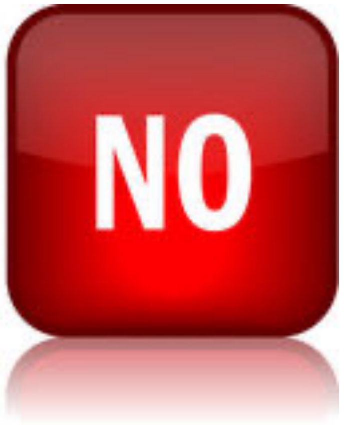

For nonvertebral fractures, the rates with and without zolpidem treatment were similar for patients younger than 65, and the difference between the rates with and without zolpidem treatment increased rapidly after age 65.

The cumulative hip fracture probability is significantly higher for zolpidem users

Fang-Yu L et al., Retrospective Population Cohort Study on Hip Fracture Risk Associated with Zolpidem Medication, Sleep 2014

6,978 patients newly prescribed for zolpidem and 27,848 nonusers between 2000-2001

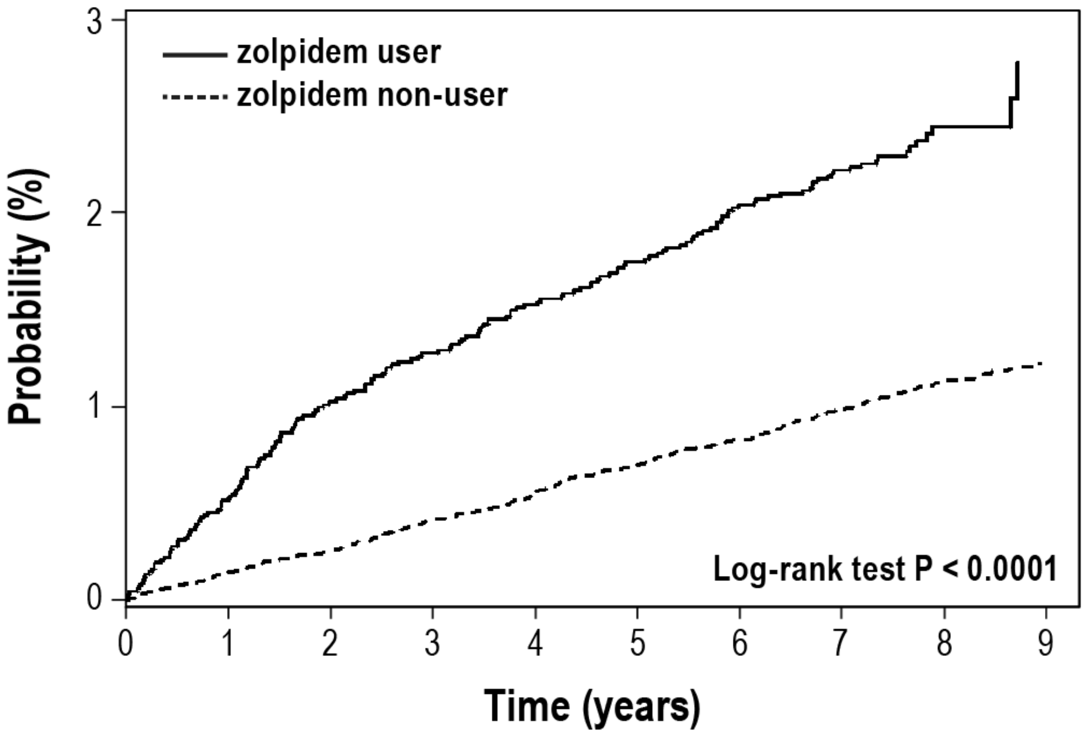

Zolpidem is not a safer alternative to BZDs in older adults

Adapted from Finkle WD et al., Risk of Fractures Requiring Hospitalization After an Initial Prescription for Zolpidem, Alprazolam, Lorazepam, or Diazepam in Older Adults, J Am Geriatr 2011

Between Jan 1, 1999 and Sept 30, 2009, Patients aged 65 and older

Fracture	Zolpidem	Alprazolam	Lorazepam	Diazepam
N	10,857	20,429	42,080	16,372
Adjusted N	-	14,939.9	29,793.8	10,150.0
**Nonvertebral**				
RR (95% CI)	**2.55** (1.78-3.65)	1.14 (0.80-1.64)	1.53 (1.23-1.91)	1.97 (1.22-3.18)
P-value	<0.001	0.42	<0.001	0.01
**Hip**				
RR (95% CI)	**3.11** (1.96-4.91)	1.46 (0.91-2.35)	2.05 (1.58-2.66)	2.03 (1.03-4.00)
P-value	<0.001	0.1	<0.001	0.04

Stilnox…. **Xanax….** **Loramet….**
Valium….

Think about non-drug treatment for insomina

Which tips is recognized for a better sleep?

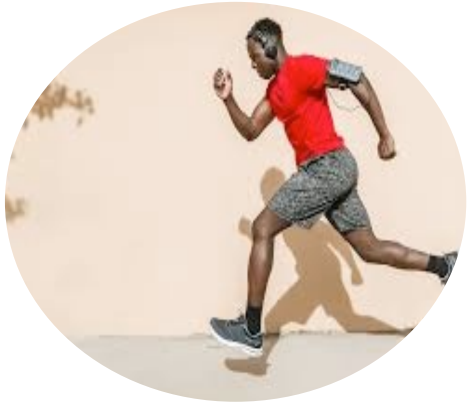

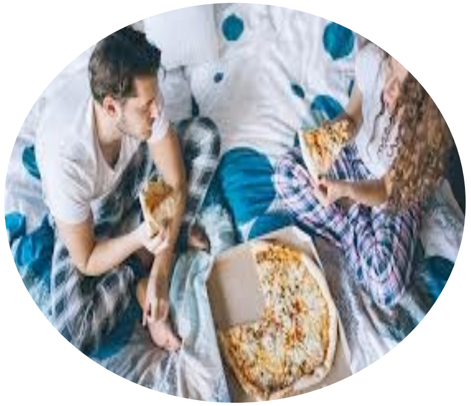

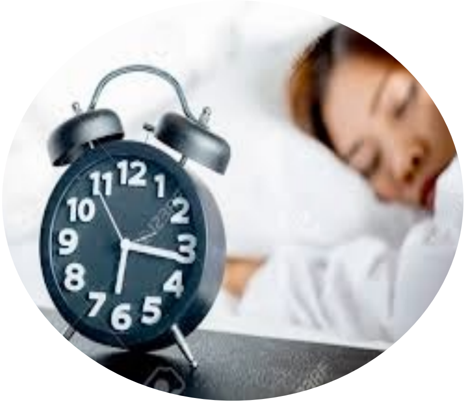

Doing more sport activities during the day

Light meal before sleeping

Keep a routine

Choose the correct answer

Valider

Tips for better sleep:

Exercise. Physical activity helps people sleep better. But avoid vigorous activity for several hours before bedtime.

Keep a routine. Try to go to bed and wake up at about the same time every day, even on weekends.

Try not to eat right before bedtime. Eat three hours or more before going to bed.

Avoid caffeine after 3 p.m. Some people need to avoid caffeine even earlier.

Limit alcohol. Alcohol causes sleepiness at first, followed by wakefulness.
Create the right environment. Keep the bedroom peaceful. And avoid mental excitement before bedtime.

Avoid bright lights. Watching a bright screen can make you stay awake.

Control pets. Pets disrupt sleep if they are on and off the bed, taking up space, or wanting to be let out.

If you don’t fall asleep soon, get out of bed and do something that will make you sleepy, such as reading. Return to bed after you start to feel drowsy.

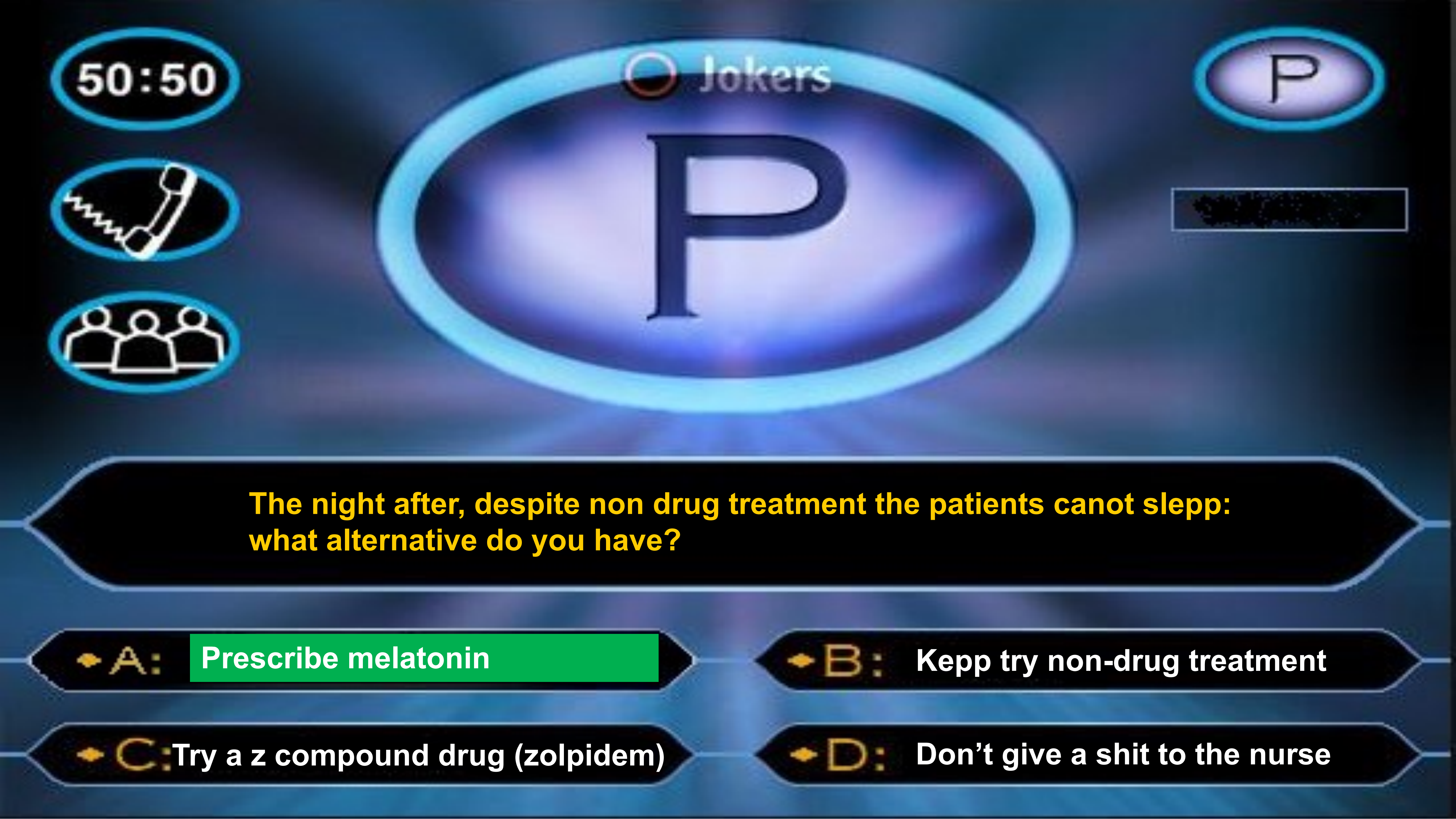
50:50
Jokers
P
P
[redacted]
The night after, despite non drug treatment the patients canot slepp: what alternative do you have?
A: Prescribe melatonin
B: Kepp try non-drug treatment
C: Try a z compound drug (zolpidem)
D: Don't give a shit to the nurse

Melatonin

- Melatonin, chemically (N-acetyl-5-methoxy-tryptamine) is a hormone secreted by pineal gland in the brain. Melatonin produced by the retina and the gastrointestinal (GI) tract acts as a paracrine hormone.

- It found in a wide spectrum of organisms including, animals, plants, bacteria and fungi. It helps regulate other hormones and maintains the body's circadian rhythm.

Melatonin Secretion

(image by Luke Mastin)

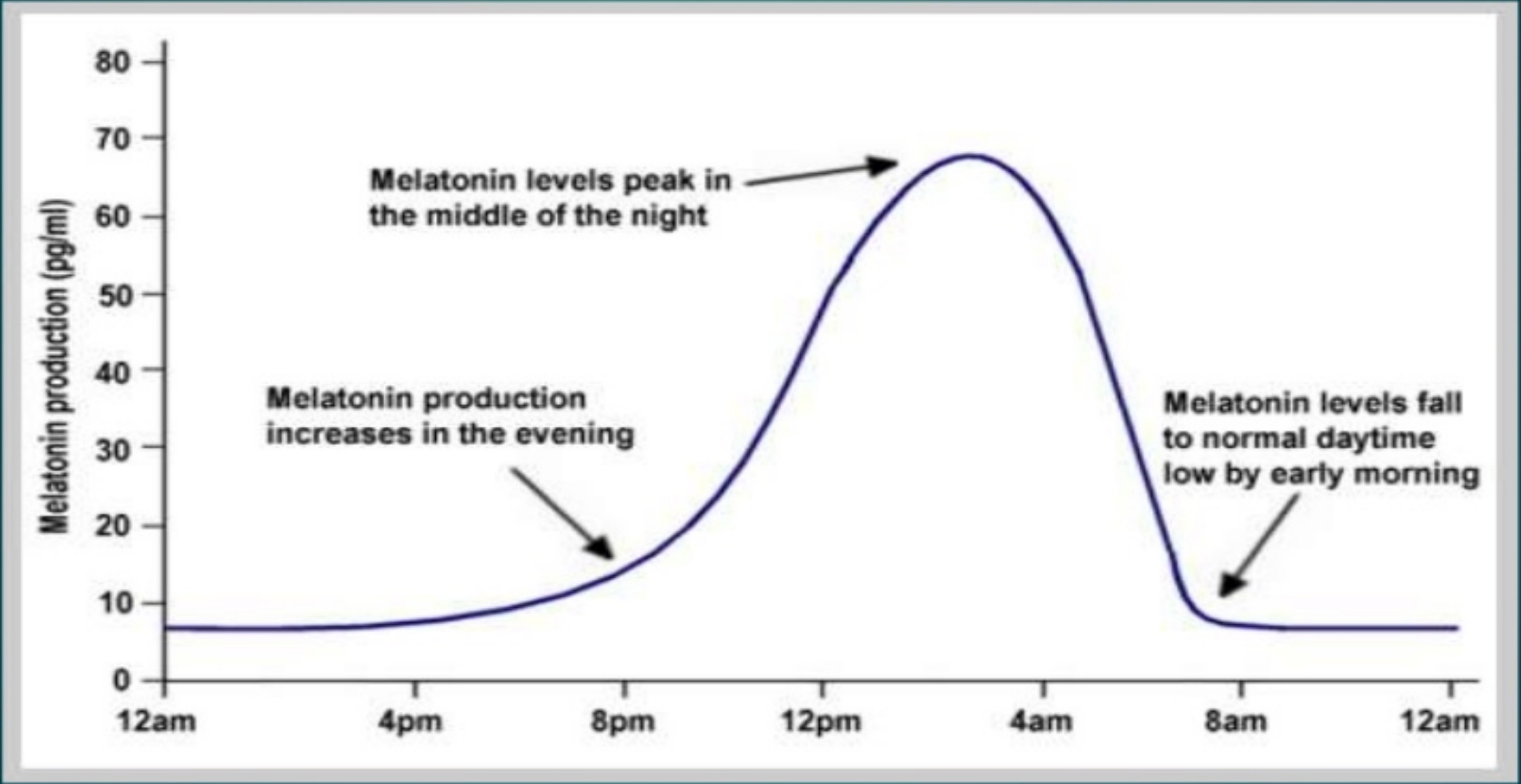

Figure 1. Sleep efficiency in subjects with normal sleep (A) and age-related insomnia (B) following melatonin or ...

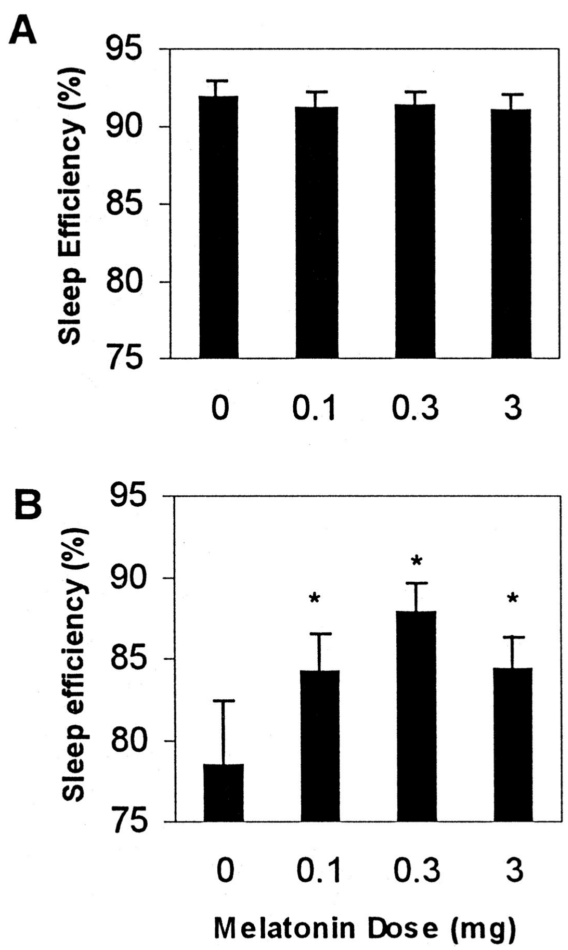

J Clin Endocrinol Metab, Volume 86, Issue 10, 1 October 2001, Pages 4727–4730, https://doi.org/10.1210/jcem.86.10.7901

The content of this slide may be subject to copyright: please see the slide notes for details.

Table 3. Patients' Self-Reports of Sleep at Baseline and Endpoint

Variable	Melatonin (N = 18)		Placebo (N = 15)		Significance
	Mean (SD)	Range	Mean (SD)	Range	
Time to fall asleep at baseline, h	1.4 (1.1)	0.3–3.0	1.8 (1.0)	0.2–3.0	NS
Time to fall asleep at endpoint, h	0.3 (0.2)	0.1–0.5	1.0 (1.0)	0.2–3.0	z = 1.96, p = .05
No. of nighttime awakenings at baseline	3.4 (1.2)	1–5	3.4 (0.9)	2–5	NS
No. of nighttime awakenings at endpoint	2.0 (0.8)	1–3	2.2 (1.3)	1–5	NS
Duration of sleep at baseline, h	3.8 (1.2)	1–5.5	3.8 (1.4)	2–6	NS
Duration of sleep at endpoint, h	5.9 (0.9)	4–7	5.0 (1.6)	2–7	t = 1.79, df = 22, p = .08
Global experience of benefit[a]	2.5 (1.0)	0–4	1.6 (1.3)	0–4	t = 2.24, df = 27, p = .03

[a]As rated on a 5-point scale, with higher ratings indicating greater benefit.

Melatonin in medically ill patients with insomnia: a double-blind, placebo-controlled study. AU Andrade C, Srihari BS, Reddy KP, Chandramma L SO J Clin Psychiatry. 2001;62(1):41.

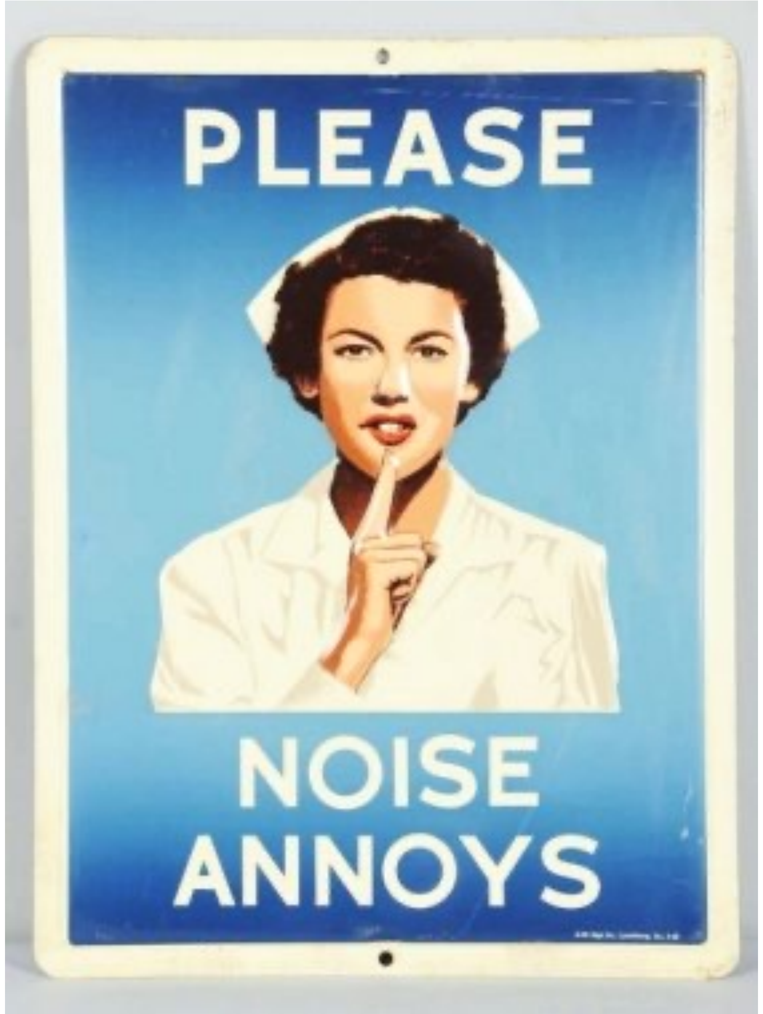

Contributing factors to sleep disorder at hospital

- **Noise at hospital (mean peak noise level of about 70 dB)**
- **Vital signs monitoring during the night**
- **Light**
- **Bed rest**

J.C. Yoder, P.G. Staisiunas, D.O. Meltzer, *et al.***Noise and sleep among adult medical inpatients: far from a quiet night**
Arch Intern Med, 172 (1) (2012), pp. 68-70
https://www.sciencedirect.com/science/article/pii/S1389945718303149#bib96

Clinical Implications and Conclusion

- **Older adults have increased sensitivity to BZDs** and decreased metabolism of long-acting agents[5].
- Despite their potential to increase in the elderly the **risk of falls, fractures, car crashes, delirium and cognitive impairment**, the use of BDZs remains high (~9%)[5,6,7].

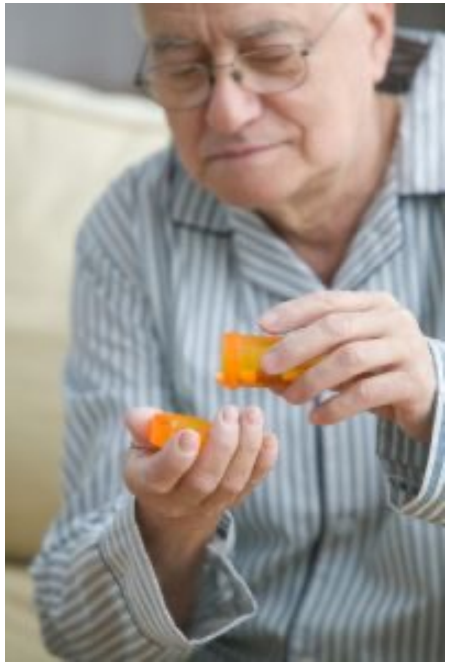

BZDs and other sedative hypnotics **are to be avoided** in the treatment of insomnia **in the elderly** because of their harms balanced with their transient efficacy.

The 2015 American Geriatrics Society Beers Criteria, J Am Geriatr Soc, 2015; 63(11):2227-46
Davidoff AJ et al., J Am Geriatr Soc 2015; 63(3):486-500
Olfson M et al., JAMA Psychiatry 2015; 72(2):136-42
