# Supplementary material for: Implementing a Multifaceted Intervention among Internal Medicine Residents with Audit and Educative Data Feedback Significantly Reduces Low-Value Care in Hospitalized Patients
Source: J Clin Med. 2022 Apr 26;11(9):2435. doi: 10.3390/jcm11092435 (PMC9104072; doi:10.3390/jcm11092435)
Supplement: Supplementary file 1 [file jcm-11-02435-s001.zip › Supplementary File S1/Vignette_smarterindicateur_PPI.pdf]

UNIVERSITÉ DE GENÈVE
FACULTÉ DE MÉDECINE

smarter medicine
Choosing Wisely Switzerland

Module E-learning

Inhibiteurs de la pompe à protons

Version 1.0 3.01.2021

Commencer

Introduction

- Les inhibiteurs de la pompe à protons (IPP) sont très souvent prescrits
- Les indications reconnues au long terme sont réduites
- La prescription au long terme est non seulement inutile mais est associée à des potentiels préjudices.

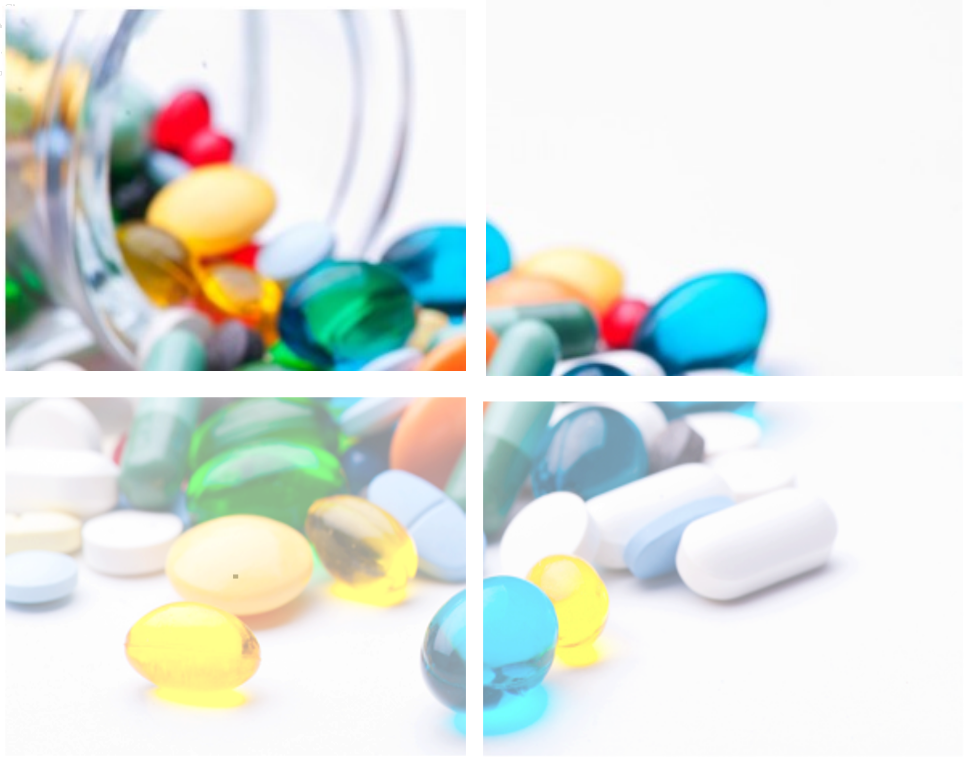

Esteves M et al Rev Med Suisse, 2017

Différents type d'IPP disponibles sur le marché

PPI Availability

PPI	Standard dose (healing) (once daily)*	Low dose (maintenance) (once daily)
Omeprazole (Losec®) - Capsule	20 mg[+]	10 mg[+]
Esomeprazole (Nexium®) - Tablet	20[a] or 40[b] mg	20 mg
Lansoprazole (Prevacid®) - Capsule	30 mg[+]	15 mg[+]
Dexlansoprazole (Dexilant®) - Tablet	30[c] or 60[d] mg	30 mg
Pantoprazole (Tecta®, Pantoloc®) - Tablet	40 mg	20 mg
Rabeprazole (Pariet®) - Tablet	20 mg	10 mg

«Principaux médicaments utilisés dans les hôpitaux Suisse

Number of prescriptions	Most frequent used products by trade-names (®)	IT code
3341	Saline 0.9%, ringer-lactate, glucose 5%	05.03.20
2943	Paracetamol	01.01.10
2037	Morphine, Fentanyl, Pethidin	01.01.30
1795	Nadroparin	06.03.30
1772	Esomeprazol, Omeprazol	04.99.00
1691	Toresamid, Furosemid	05.01.00
1456	Sodium-picosulfate, Sodium-dihydrogenophosphate	04.08.11
1245	Quinapril, Valsartan	02.07.10
1216	Amoxicillin, Cotrimoxazol, Piperacilin Tazobactam	08.01.93
1153	Acetysalicylic acid	06.03.20
1121	Lorazepam	01.04.10
885	Valerian extract	01.04.20
847	Salbutamol, Ipratropiumbromide	03.04.30
671	Atorvastatin	07.12.00
667	Bisoprolol, Metoprolol	02.03.00
643	Ciprofloxacin, Moxifloxacin	08.01.80
614	Zolpidem, Midazolam, Triazolam	01.30.10
560	Felodipin, Amlodipin	02.06.10
535	Metformin, Gliclazid, Pioglitazon	07.06.20
463	Phenprocoumon	06.03.10
459	Acetylcystein	03.02.00
423	Citalopram, Paroxetin, Clomipramin	01.06.00
335	Dexpanthenol	12.02.40
334	Cefepime	08.01.30
312	Sacharmyces	04.09.00

Indications reconues aux IPP

Dans certaines situations cliniques, les IPP sont indiqués au long cours (>8sem).

Q.2: Quels sont selon vous ces situations?

Cliquez sur la/les bonne(s) réponse(s). Plusieurs réponses possibles

Œsophagite sévère

Ulcère aux AINS

Œsophage de Barrett

En prévention chez patients à risque sous AINS

Cliquez sur les bonnes réponses

Indications reconues aux IPP

Dans certaines situations cliniques, les IPP sont indiqués au long cours (>8sem).

Q.2: Quels sont selon vous ces situations?

Cliquez sur la/les bonne(s) réponse(s). Plusieurs réponses possibles

Œsophagite sévère

Ulcère aux AINS

Œsophage de Barrett

En prévention chez patients à risque sous AINS

Cliquez sur les bonnes réponses

Savoir reconnaître les indications appropriées aux IPP

Table 1 Summary of the conclusions by Scarpignato et al. [3] regarding the appropriateness of proton pump inhibitor (PPI) therapy in 13 clinical scenarios of uncertainty and common misuse

	Reason for use
Long-term PPI therapy appropriate	• Barrett's esophagus<br>• Healing and maintenance of healed Los Angeles grade C or D erosive esophagitis[a]<br>• PPI-responsive esophageal eosinophilia<br>• Idiopathic (*H. pylori* and NSAID/aspirin negative) peptic ulcer disease<br>• Zollinger–Ellison disease[a,b]<br>• PPI-responsive GERD/non-erosive reflux disease[a,c]<br>• Long-term non-selective NSAID users at high-risk for upper GI complications or long-term cox-2 inhibitor users with a prior episode of GI bleeding[a]<br>• Anti-platelet therapy in patients at high-risk for upper GI complications (age > 65 years or concomitant use of corticosteroids or anticoagulants or history of peptic ulcer disease)<br>• Steatorrhea refractory to enzyme replacement therapy in chronic pancreatitis
Short-term PPI therapy appropriate (4- to 12-week course)	• Healing of Los Angeles grade A or B erosive esophagitis[a]<br>• Eosinophilic esophagitis<br>• *H. pylori* eradication (in combination with antibiotics)[a,d]<br>• Stress ulcer prophylaxis in high-risk patients (i.e., critically ill patients with respiratory failure or coagulopathy)<br>• Functional dyspepsia<br>• Treatment and maintenance of peptic ulcer disease[a]<br>• Prior to endoscopy for acute upper GI bleeding<br>• Following endoscopic treatment of a high-risk ulcer GI bleed
PPI use not appropriate	• Corticosteroid users without concomitant NSAID therapy<br>• To prevent bleeding from hypertensive gastropathy in cirrhotic patients<br>• Acute pancreatitis<br>• Stress ulcer prophylaxis in non-critically ill hospitalized patients that are not at high-risk for ulcer formation and GI bleeding
PPI use of uncertain benefit	• PPI non-responsive GERD<br>• Extra-digestive GERD

[a]FDA approved indications
[b]Requires 3–4 times the usual dose (PPI therapy is typically started as single dose)
[c]In these cases, a PPI taper should be attempted to the lowest effective dose, on demand dosing, or intermittent dosing
[d]In this case, a 1 to 2 week course of PPI therapy for *H. pylori* eradication in conjunction with antibiotics is appropriate
GERD gastroesophageal reflux disease, *GI* gastrointestinal, *NSAID* non-steroidal anti-inflammatory drugs

Scarpignato et al. BMC Medicine 2016

Indications reconnues des IPP

Reflux gastro-eosophagien (GERD/NERD)

Long terme

- Oesophagite sévère ou Barrett
- Persistence des symptômes:
  - Maintenir la dose minimale efficace
  - Traitement à la demande

Hémorragie digestive haute

- Court terme: haut risque→ haute dose 72h
- Moyen terme:
  - Ulcère duodenal/estomac
    - IPP 4-8 sem dose standard

      Rockall >6 = double dose 11 jours puis dose standard
- Varice oesophagienne IPP 10j dose standard

Prévention ulcère NSAID/AAS

- IPP si risque élevé (atcd ulcère ou ≧ 3 facteurs de risque)
- Pas besoin IPP si corticoïde uniquement

Facteurs de risque

Age >65 ans

Haute dose AINS

Prise concommitante (AAS, antiplaquettaire, Cortisone, ACO, SSRI?)

Complications des IPP

Quels sont les effets secondaires associés avec la prescription des IPP

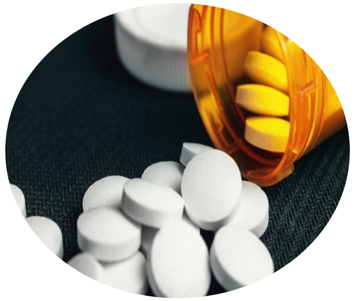

Addiction

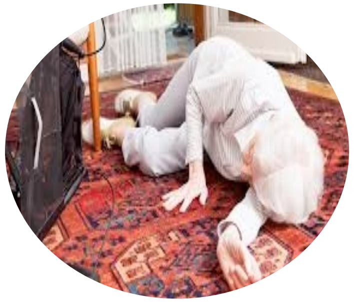

Fracture

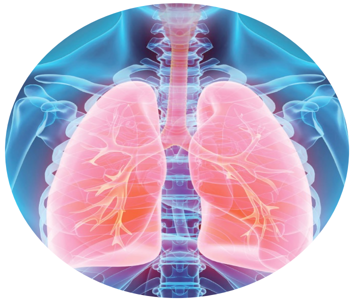

Colite infectieuse

Choisissez la bonne réponse

Valider

Complications des IPP

Quels sont les effets secondaires associés avec la prescription des IPP

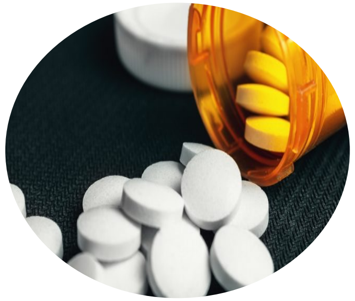

Addiction

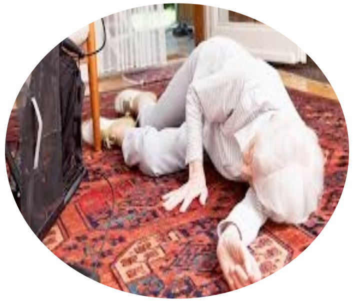

Fracture

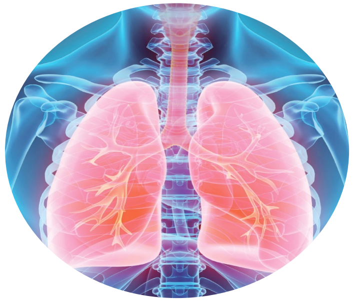

Colite infectieuse

Choisissez la bonne réponse

Valider

Complications des IPP au long cours

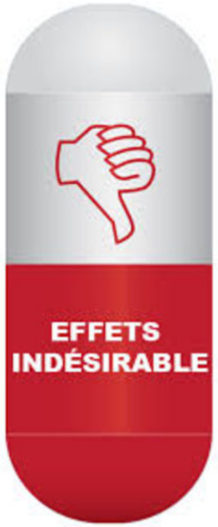

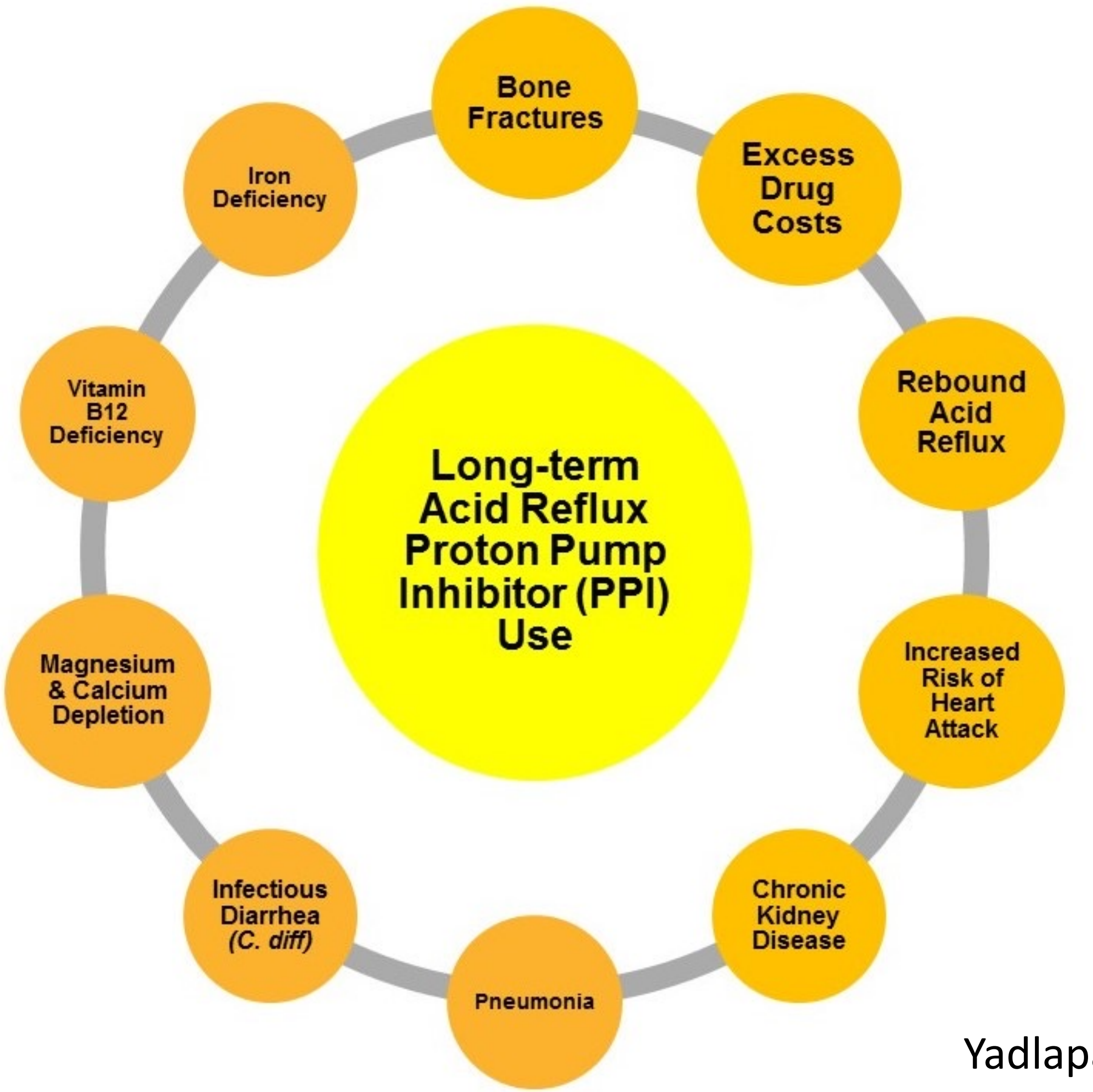

Yadlapati et al. BMC med 2017

Gastroenterology 2019;157:682–691

Safety of Proton Pump Inhibitors Based on a Large, Multi-Year, Randomized Trial of Patients Receiving Rivaroxaban or Aspirin

Paul Moayyedi,[1] John W. Eikelboom,[1] Jackie Bosch,[1] Stuart J. Connolly,[1] Leanne Dyal,[1]

Table 3. Other Prespecified Safety Outcomes

	Incident events, n (%)		Pantoprazole, 40 mg od, vs placebo	
Outcome	Pantoprazole, 40 mg od (n = 8791)	Placebo (n = 8807)	OR (95% CI)	*P* value
Gastric atrophy	19 (0.2)	26 (0.3)	0.73 (0.40–1.32)	.30
Clostridium difficile	9 (0.1)	4 (<0.1)	2.26 (0.70–7.34)	.18
Other enteric infection	119 (1.4)	90 (1.0)	1.33 (1.01–1.75)	.04
Chronic kidney disease	184 (2.1)	158 (1.8)	1.17 (0.94–1.45)	.15
Dementia	55 (0.6)	46 (0.5)	1.20 (0.81–1.78)	.36
Pneumonia	318 (3.6)	313 (3.6)	1.02 (0.87–1.19)	.82
Fracture	203 (2.3)	211 (2.4)	0.96 (0.79–1.17)	.71
COPD	146 (1.7)	124 (1.4)	1.18 (0.93–1.51)	.17
Diabetes mellitus	513 (5.8)	532 (6.0)	0.96 (0.85–1.09)	.56

COPD, chronic obstructive pulmonary disease; od, once daily.

Raison des prescriptions inappropriées

A votre avis, quelles sont les raisons de ces prescriptions inappropriées?

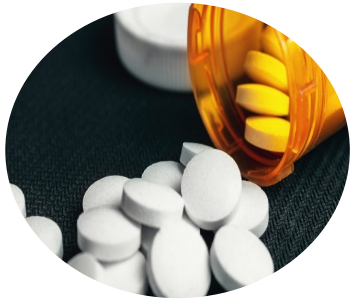

Méconnaissance des indications

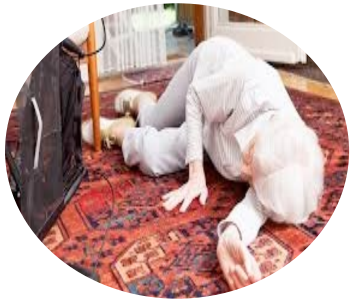

Effet rebond

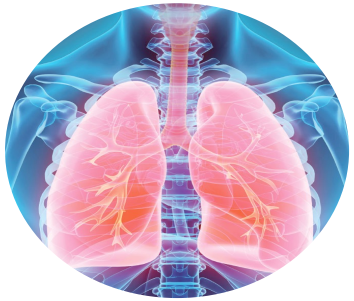

Non arrêt lors des transitions de soins

Choisissez les bonnes réponses

Valider

Raison des prescriptions inappropriées

A votre avis, quelles sont les raisons de ces prescriptions inappropriées?

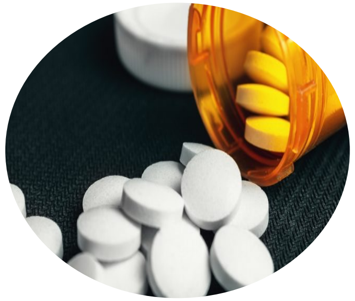

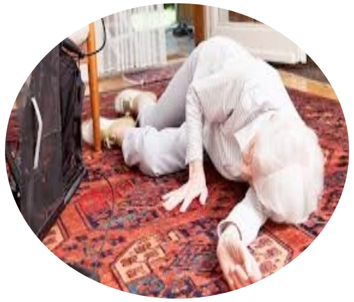

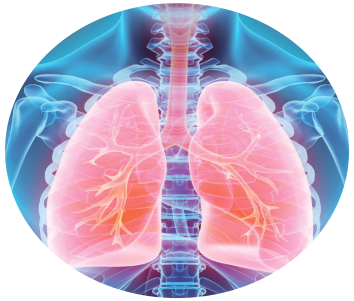

Méconnaissance des indications

Effet rebond

Non arrêt lors des transitions de soins

Choisissez les bonnes réponses

Valider

1. Mauvaise indication

Table 1 Summary of the conclusions by Scarpignato et al. [3] regarding the appropriateness of proton pump inhibitor (PPI) therapy in 13 clinical scenarios of uncertainty and common misuse

	Reason for use
Long-term PPI therapy appropriate	• Barrett's esophagus<br>• Healing and maintenance of healed Los Angeles grade C or D erosive esophagitis[a]<br>• PPI-responsive esophageal eosinophilia<br>• Idiopathic (*H. pylori* and NSAID/aspirin negative) peptic ulcer disease<br>• Zollinger–Ellison disease[a,b]<br>• PPI-responsive GERD/non-erosive reflux disease[a,c]<br>• Long-term non-selective NSAID users at high-risk for upper GI complications or long-term cox-2 inhibitor users with a prior episode of GI bleeding[a]<br>• Anti-platelet therapy in patients at high-risk for upper GI complications (age > 65 years or concomitant use of corticosteroids or anticoagulants or history of peptic ulcer disease)<br>• Steatorrhea refractory to enzyme replacement therapy in chronic pancreatitis
Short-term PPI therapy appropriate (4- to 12-week course)	• Healing of Los Angeles grade A or B erosive esophagitis[a]<br>• Eosinophilic esophagitis<br>• *H. pylori* eradication (in combination with antibiotics)[a,d]<br>• Stress ulcer prophylaxis in high-risk patients (i.e., critically ill patients with respiratory failure or coagulopathy)<br>• Functional dyspepsia<br>• Treatment and maintenance of peptic ulcer disease[a]<br>• Prior to endoscopy for acute upper GI bleeding<br>• Following endoscopic treatment of a high-risk ulcer GI bleed
PPI use not appropriate	• Corticosteroid users without concomitant NSAID therapy<br>• To prevent bleeding from hypertensive gastropathy in cirrhotic patients<br>• Acute pancreatitis<br>• Stress ulcer prophylaxis in non-critically ill hospitalized patients that are not at high-risk for ulcer formation and GI bleeding
PPI use of uncertain benefit	• PPI non-responsive GERD<br>• Extra-digestive GERD

[a]FDA approved indications
[b]Requires 3–4 times the usual dose (PPI therapy is typically started as single dose)
[c]In these cases, a PPI taper should be attempted to the lowest effective dose, on demand dosing, or intermittent dosing
[d]In this case, a 1 to 2 week course of PPI therapy for *H. pylori* eradication in conjunction with antibiotics is appropriate
GERD gastroesophageal reflux disease, *GI* gastrointestinal, *NSAID* non-steroidal anti-inflammatory drugs

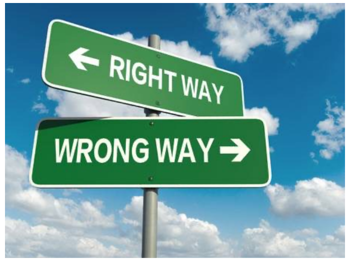

2. Poursuite des IPP pendant transition des soins

RESEARCH

Prevalence of potentially inappropriate prescribing in older people in primary care and its association with hospital admission: longitudinal study

Teresa Pérez,[1,2] Frank Moriarty,[2] Emma Wallace,[2] Ronald McDowell,[2,3] Patrick Redmond,[2,4] Tom Fahey[2]

- 10% des admissions >65ans: effets indésirables des médicaments
- Admission à l'hôpital associée à un risque de 72 % d'avoir une prescription inappropriée à la sortie

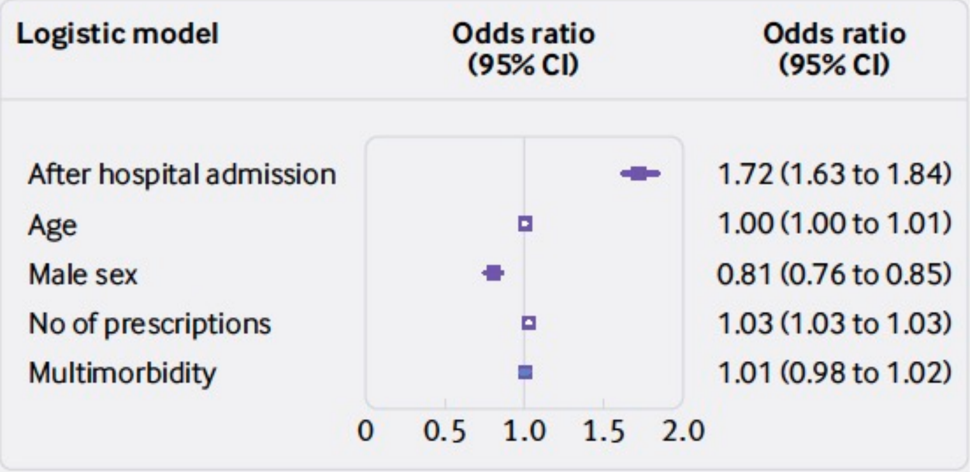

thebmj | BMJ 2018;363:k4524 | doi: 10.1136/bmj.k4524

3. Effet de rebond

Proton-Pump Inhibitor Therapy Induces Acid-Related Symptoms in Healthy Volunteers After Withdrawal of Therapy

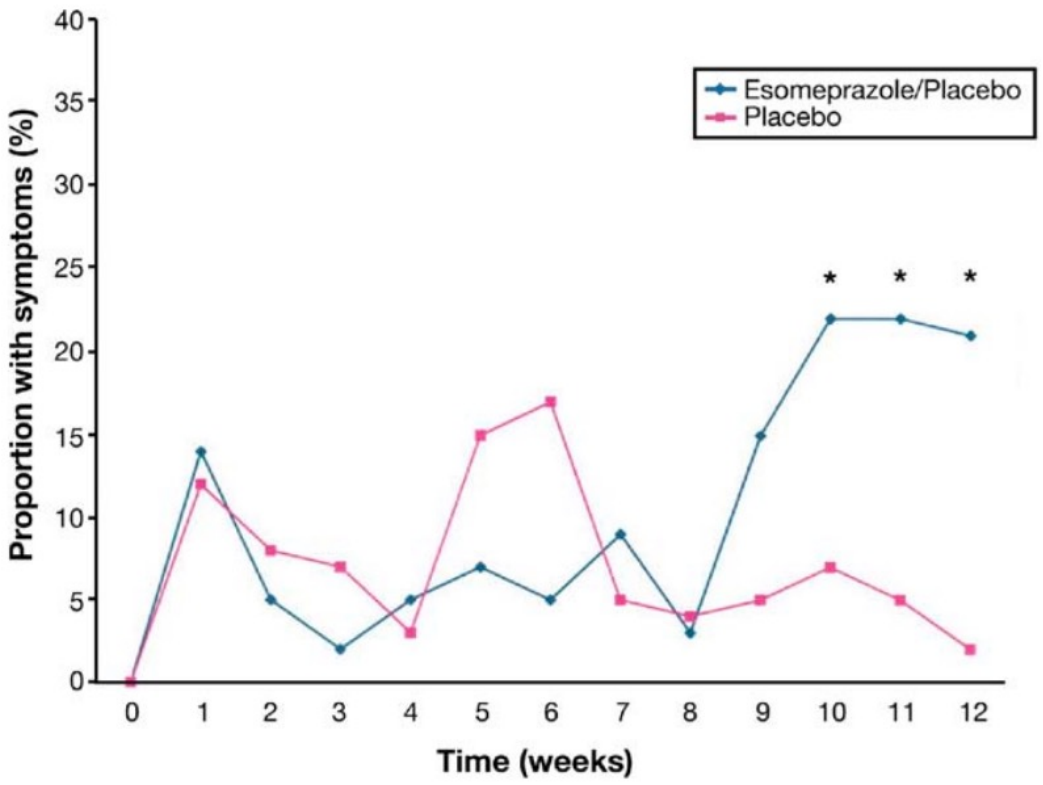

- Les patients se plaignent souvent de récidive des symptoms à l'arrêt des IPP
- L'arrêt du traitement chez des volontaires sains a provoqué des symptoms de reflux
- Le sevrage des IPP devrait se faire de manière progressive

Reimer C et al. Gastroenterology 2009

Impact Clinique et conclusion

- Les IPP font partie des médicaments les plus souvent prescrits de manière inappropriée
- Les indications aux IPP > 8sem sont rares et bien définies.
- Les IPP sont associés à des effets secondaires non négligeables telles que les colites à Clostridium
- La plus petite dose efficace doit toujours être recherché et le sevrage devrait se faire manière progressive pour éviter l’effet rebond

DEPRESCRIPTION

Choosing Wisely Canada DIY

Don't maintain long-term Proton Pump Inhibitor (PPI) therapy for gastrointestinal symptoms without an attempt to stop / reduce PPI at least once per year in most patients.

Walsh K, et al. Deprescribing in a Family Health Team:. J Prim Health Care. 2016

PIMCheck
Potentially Inappropriate Medication - Patients in the Internal Medicine unit

HUG Hôpitaux Universitaires Genève

https://www.pimcheck.org/en/presentation.php

Campagne publicitaire « Choosing wisely » pour éviter la prescription inappropriée de IPP

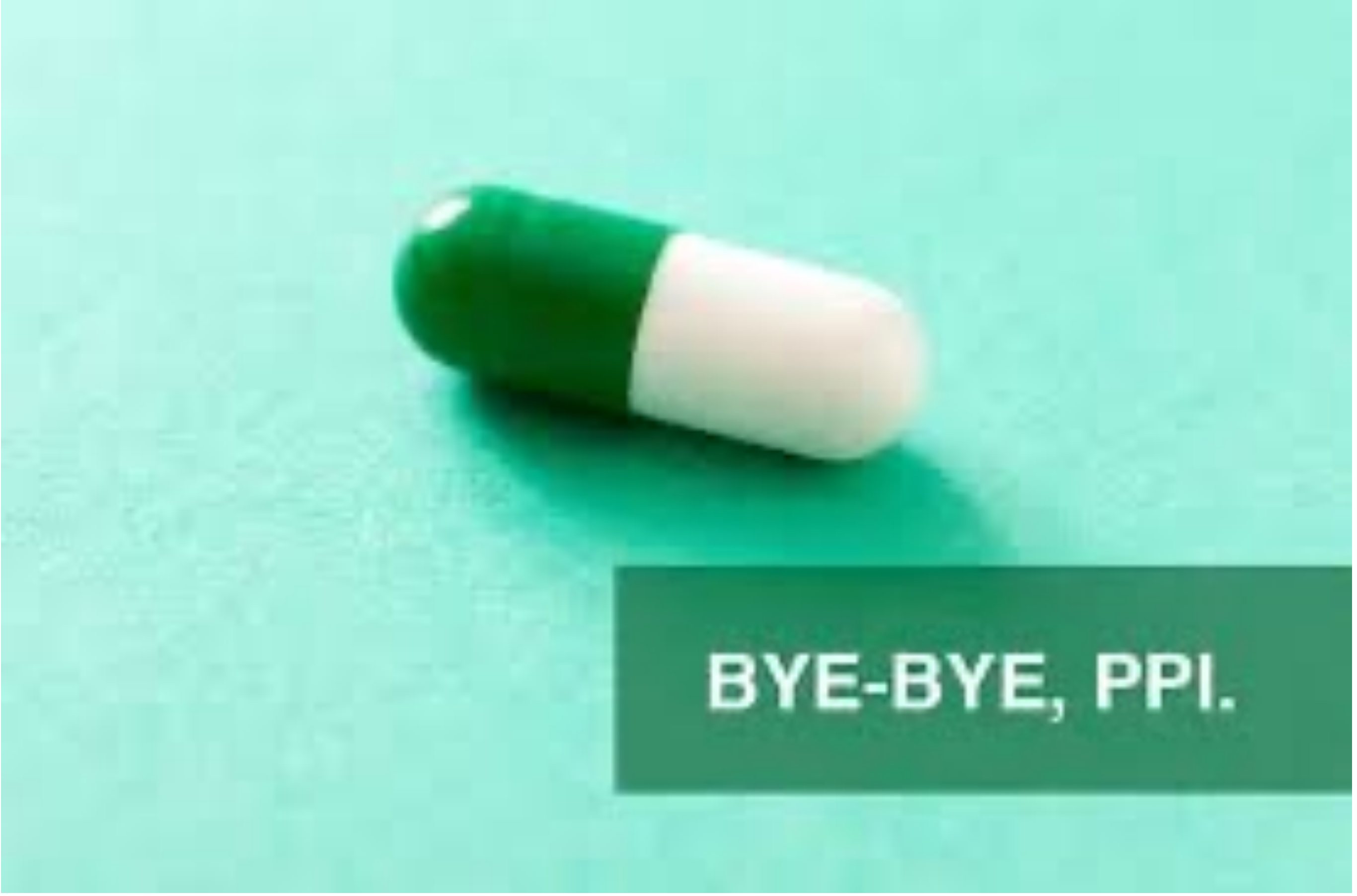

Toujours diminuer à la plus petite dose efficace

Choosingwisely.org
