# Supplementary material for: Implementing a Multifaceted Intervention among Internal Medicine Residents with Audit and Educative Data Feedback Significantly Reduces Low-Value Care in Hospitalized Patients
Source: J Clin Med. 2022 Apr 26;11(9):2435. doi: 10.3390/jcm11092435 (PMC9104072; doi:10.3390/jcm11092435)
Supplement: Supplementary file 1 [file jcm-11-02435-s001.zip › Supplementary File S1/Vignette_smarterindicateur_prisedesang.pdf]

UNIVERSITÉ DE GENÈVE
FACULTÉ DE MÉDECINE

smarter medicine
Choosing Wisely Switzerland

Module E-learning

Prélèvements sanguins

Version 1.0 17.12.2020

Commencer

Introduction

- De nombreux examens de laboratoire sont demandés à intervalles réguliers (parfois quotidiennement)
- Un examen de laboratoire devrait être demandé uniquement pour répondre à une question clinique précise
- La prescription régulière des tests augmente les coûts de la santé, ne profite pas aux patients et peut en fait leur nuire.

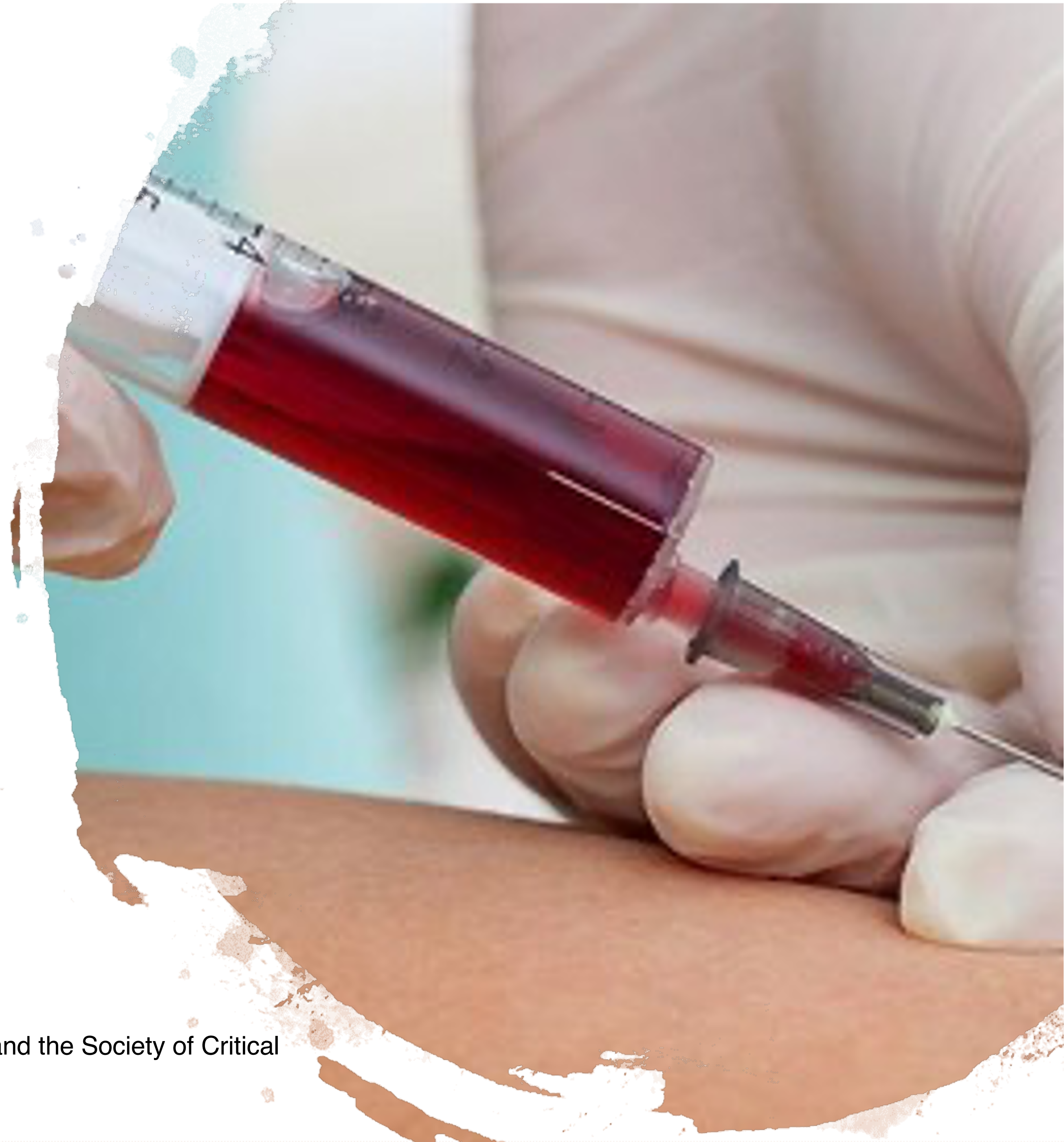

Sources : the American College of Chest Physicians, the American Thoracic Society and the Society of Critical Care Medicine & CH expert panel

Inconvénients des prises de sang

On comprend aisément que les prises de sang permettent d'orienter le diagnostic clinique bien que peu d'études l'aient scientifiquement démontré.

Q.2: Quels sont selon vous les éventuelles complications liées à aux prises de sang parmi ces propositions?

Cliquez sur la/les bonne(s) réponse(s). Plusieurs réponses possibles

Douleur

Faux positifs

Dracula syndrome

Réaction anaphylactique

Cliquez sur les bonnes réponses

Inconvénients des prises de sang

On comprend aisément que les prises de sang permettent d'orienter le diagnostic clinique bien que peu d'études l'aient scientifiquement démontré.

Q.2: Quels sont selon vous les éventuelles complications liées à aux prises de sang parmi ces propositions?

Cliquez sur la/les bonne(s) réponse(s). Plusieurs réponses possibles

- Douleur
- Faux positifs
- Dracula syndrome
- Réaction anaphylactique

Alonso-Cerezo MC et al., Clin Chem Lab Med 2009; 47(12):1461-5
Detsky AS and Krumholz HM, JAMA 2014; 311(21):2169-70

Inconvénients des prises de sang de routine

- Douleur et incomfort lors du prélèvement, y compris l'aspect psychologique
- Risque de générer des faux positifs
  - Cascade d'examens complémentaires et traitement inutiles
- "Dracula syndrome"

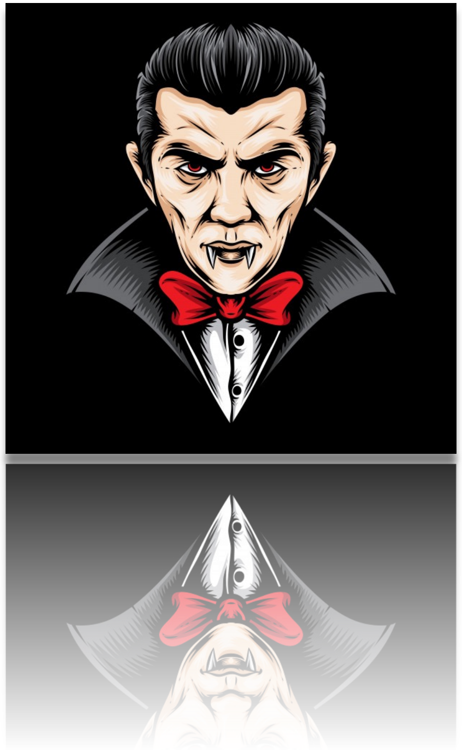

Les prélèvements répétés de sang diminuent les valeurs d'hémoglobine et peuvent résulter à une anémie nosocomiale, elle-même associée à un prolongement des durées de séjour et le besoin de transfusion de sang.

- Coûts non nécessaires

Alonso-Cerezo MC et al., Clin Chem Lab Med 2009; 47(12):1461-5
Detsky AS and Krumholz HM, JAMA 2014; 311(21):2169-70

ORIGINAL INVESTIGATION

ONLINE FIRST | LESS IS MORE

Arch Intern Med. 2011;171(18):1646-1653.
Published online August 8, 2011.
doi:10.1001/archinternmed.2011.361

Diagnostic Blood Loss From Phlebotomy and Hospital-Acquired Anemia During Acute Myocardial Infarction

Adam C. Salisbury, MD, MSc; Kimberly J. Reid, MS; Karen P. Alexander, MD; Frederick A. Masoudi, MD, MSPH; Sue-Min Lai, PhD, MS, MBA; Paul S. Chan, MD, MSc; Richard G. Bach, MD; Tracy Y. Wang, MD, MHS, MSc; John A. Spertus, MD, MPH; Mikhail Kosiborod, MD

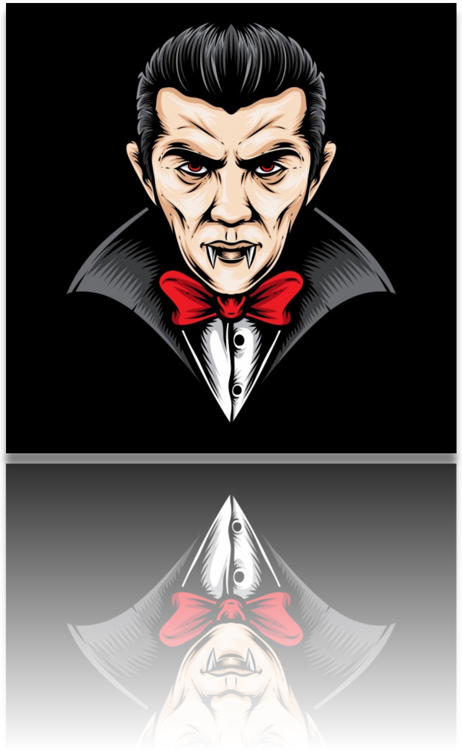

20% of patients with acute MI develop moderate to severe hospital-acquired anemia (Hb <11 g/dl):

63 ml for chemistry
39 ml for hematology
29 ml for coagulation studies
20 ml for blood cultures
20 ml for ABGA

Variation des soins selon différents hôpitaux sur le nombre de ml de prélèvement de sang

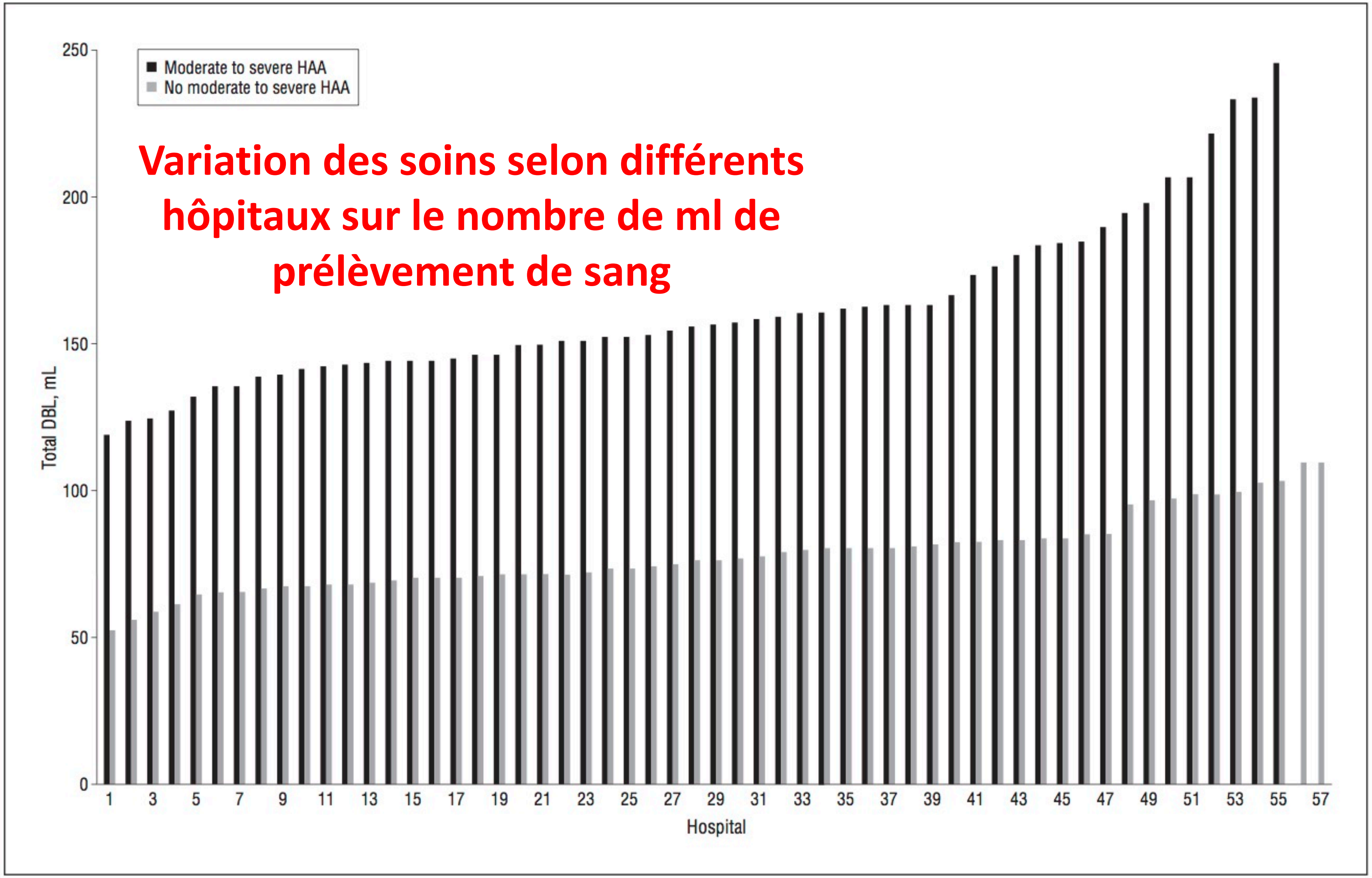

Figure 2. Variation in mean diagnostic blood loss (DBL) across the 57 hospitals included in a contemporary acute myocardial infarction database (Cerner Corp's Hospital Facts database.) Bars represent shrinkage estimates of the mean DBL for patients' entire hospitalizations across each hospital. Black bars represent the mean value for patients with moderate to severe hospital-acquired anemia (HAA), and gray bars present the mean value for patients without moderate to severe HAA. Hospitals are plotted on the x-axis from the hospital with the smallest mean blood loss to the hospital with the largest, ranked separately among those with moderate to severe HAA and without moderate to severe HAA.

Q.1: Face à un patient de 54 ans anticoagulé par Xarelto pour une FA qui vient pour une une pneumonie, quel dosage sanguin effectuez-vous pour évaluer la coagulation?

Cliquez sur la bonne réponse

TP

PTT

antiXa

Aucun test

Q.1: Face à un patient de 54 ans anticoagulé par Xarelto pour une FA qui vient pour une une pneumonie, quel dosage sanguin «de routine» effectuez-vous pour évaluer la coagulation?

Cliquez sur la bonne réponse

- TP
- PTT
- antiXa
- Aucun test

Texte en pop

Chez un patient anticoagulé par un anticoagulant direct (ACOD), il est inutile de doser les facteurs de la coagulations classiques en dehors d’une situations hémorragique.

La réponse “aucun test est correct” le patient ne présentant pas de situation hémorragique

PT = Prothrombin Time
INR = International Normalized Ratio
aPTT = Activated Partial Thromboplastin Time

- Warfarin Therapy
- Liver Disease
- Risk factor for vitamin K deficiency (e.g. malnutrition, fat soluble vitamin malabsorption, cholestasis, prolonged antibiotics)

CONSIDER PT/INR

- IV Heparin monitoring
- IV Argatroban monitoring
- Suspected Hemophilia A/B, Factor XI deficiency, severe von Willebrand disease

CONSIDER aPTT

- Bleeding patient

CONSIDER BOTH PT/INR & aPTT

TOP 5 REASONS NOT to ORDER PT/INR or aPTT

1. As routine blood work.
2. As a routine pre-op screen in a patient without a personal/family bleeding history.
3. For monitoring of direct oral anticoagulant (DOAC) therapy (e.g. dabigatran, rivaroxaban, apixaban).
4. For monitoring of low molecular weight heparin (LMWH) therapy (e.g. dalteparin, enoxaparin, tinzaparin, fondaparinux).
5. For monitoring of thromboprophylaxis (e.g. heparin 5000 U SC BID; dalteparin 5000 U SC QD).

Choosingwisely.org

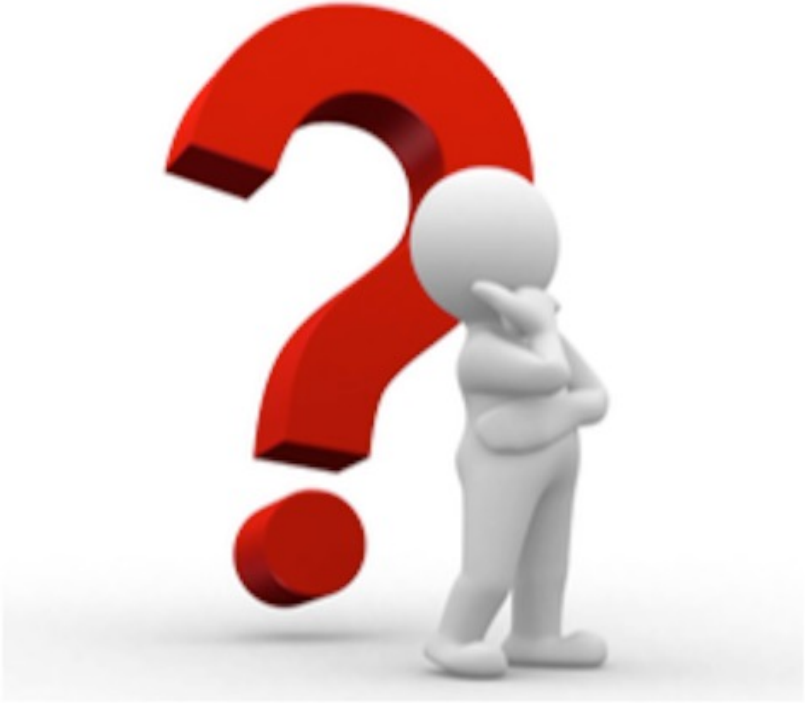

La prochaine fois, demandez-vous si votre prise de sang est vraiment nécessaire?

Va-t-elle modifier la prise en charge?

Comment faire pour modifier votre comportement?

Education pour modifier le comportement de prescrpition

- Active educational intervention (eg. interactive didactic presentations, discussions, educational flyers, weekly email communications)

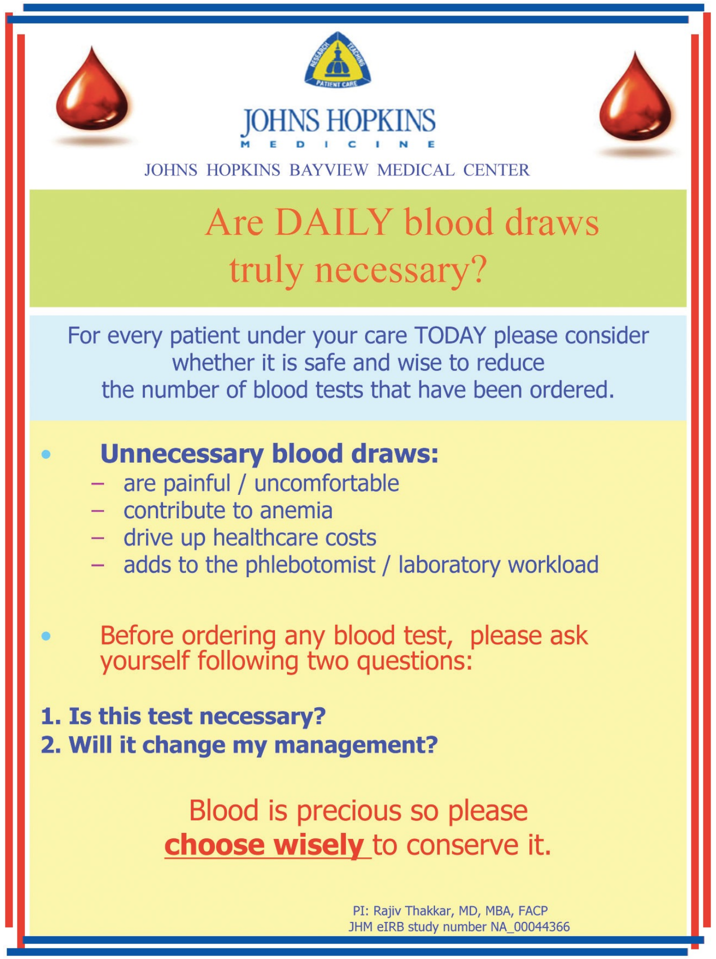

Interventions pour modifier le comportment de prescription

- Interventions éducatives (ex. Présentation intéractives didactiques, discussions, flyers, emails et utilisation de tableaux de bord)
- Audit régulier et feedback des performances des precripteurs
- Outils électroniques pour des aides à la decision clinique
- Dévelopement de guidelines pour l'utilisation appropriée des tests sanguins

Thakkar RN et al., Am J Clin Pathol 2015; 143(3):393-7

Interventions pour modifier le comportment de prescription: exemple du Triemli à ZH

- Regular audit and feedback of performance data

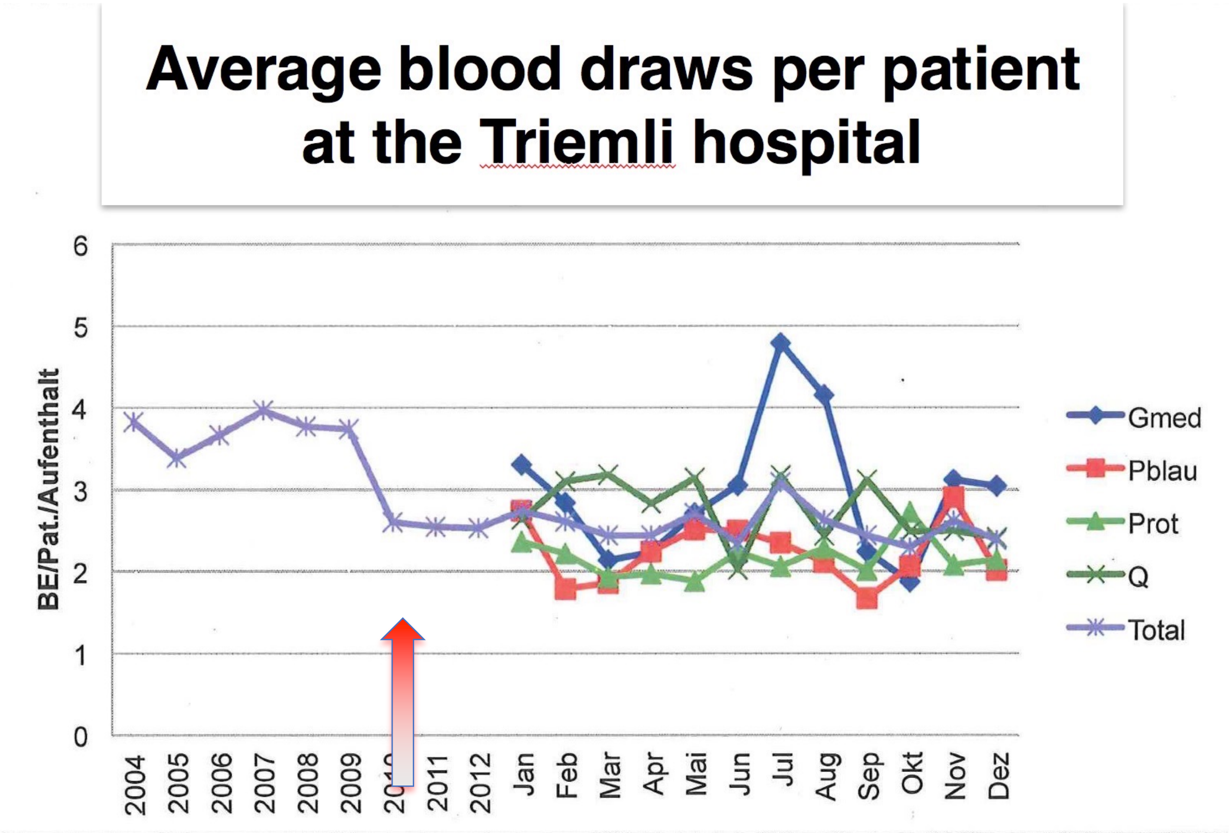

C Meier, SSMIG 2016

Impact of an education and multilevel social comparison–based intervention bundle on use of routine blood tests in hospitalised patients at an academic tertiary care hospital: a controlled pre-intervention post-intervention study

Anshula Ambasta [1], Irene Wai Yan Ma,[1] Stephen Woo,[2] Kevin Lonergan,[3] Elizabeth Mackay,[2] Tyler Williamson[1]

BMJ

Ambasta A, *et al*. *BMJ Qual Saf* 2020;**0**:1–8. doi:10.1136/bmjqs-2019-010118

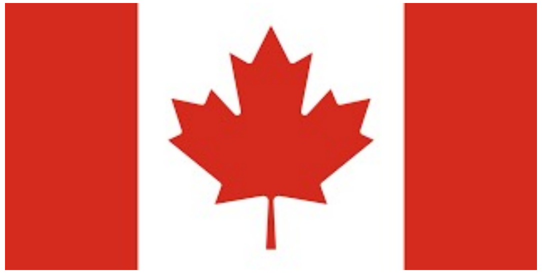

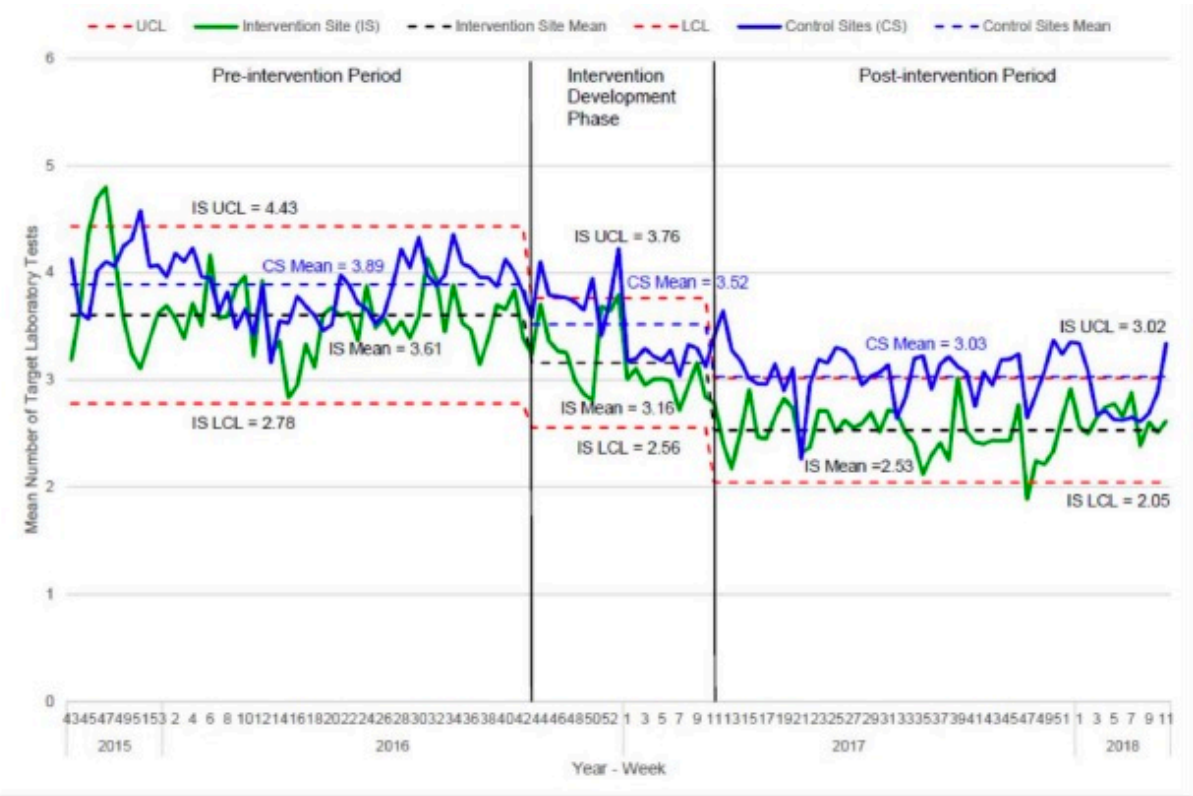

Figure 1 X-Bar control chart of the mean number of target laboratory tests per patient-day on a weekly basis during study period at Intervention and control sites. Pre-intervention period: 23 Oct 2015 to 23 Oct 2016. Intervention development phase: 24 Oct 2016 to 12 Mar 2017 (please see online supplementary appendix figure 1 for detailed sequential introduction of intervention components). Post-intervention period: 13 Mar 2017 to 13 Mar 2018. The entire intervention bundle was simultaneously implemented at the beginning of the post-intervention period, ie, 13 Mar 2017. LCL, lower control limit; UCL, upper control limit.

- incidence rate ratio=0.89; 95% CI 0.79 to 1.00; p=0.048)
- costs savings of $C68 877 (p=0.020)

Impact Clinique et conclusion

- Un examen de laboratoire devrait être demandé uniquement pour répondre à une question clinique précise
- La prescription régulière des tests augmente les coûts de la santé, ne profite pas aux patients et peut en fait leur nuire.
- Parmi ces complication, l'anémie nosocmiale "Dracula syndrome" compte parmi les complications la plus fréquente
- Des interventions pédagogiques avec un feedback éducatif permettent de réduire les prescriptions inappropriées de prises de sang.

Campagne publicitaire « Choosing wisely » pour éviter les prises de sang répétées et ainsi le risque d'anémie

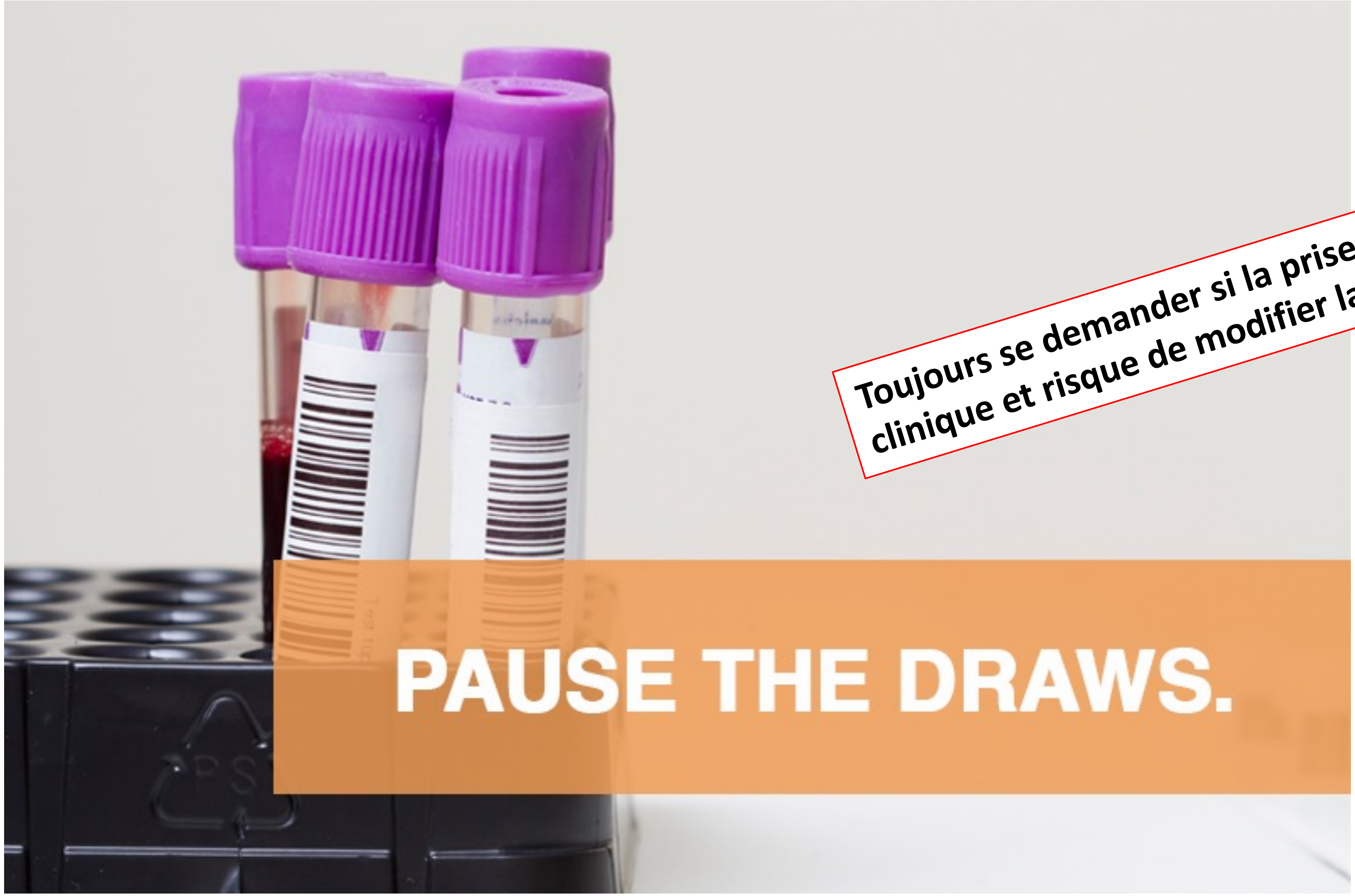

Toujours se demander si la prise de sang répond à question clinique et risque de modifier la prise en charge du patient

Choosingwisely.org
