# Supplementary material for: Implementing a Multifaceted Intervention among Internal Medicine Residents with Audit and Educative Data Feedback Significantly Reduces Low-Value Care in Hospitalized Patients
Source: J Clin Med. 2022 Apr 26;11(9):2435. doi: 10.3390/jcm11092435 (PMC9104072; doi:10.3390/jcm11092435)
Supplement: Supplementary file 1 [file jcm-11-02435-s001.zip › Supplementary File S2/HDTL_Affiche_benzodiazépines_A4.pdf]

Hôpital de La Tour

Réduire l'utilisation des benzodiazépines

Le saviez-vous ?

20 – 30 %

Les benzodiazépines sont trop souvent prescrites en milieu hospitalier

Les indications appropriées sont rares (sevrage OH, trouble anxieux, soins palliatifs)

Les benzodiazépines doivent être évitées pour les troubles du sommeil

Il existe des alternatives pour les troubles du sommeil (mélatonine, hygiène de sommeil, réduction des nuisances sonores)

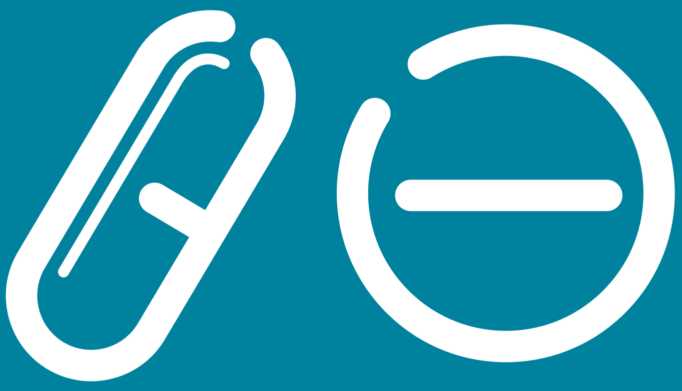

Complications

- Chute
- Fracture
- Prolongation du séjour hospitalier
- Dépendance
- Dépression respiratoire
