# Supplementary material for: Implementing a Multifaceted Intervention among Internal Medicine Residents with Audit and Educative Data Feedback Significantly Reduces Low-Value Care in Hospitalized Patients
Source: J Clin Med. 2022 Apr 26;11(9):2435. doi: 10.3390/jcm11092435 (PMC9104072; doi:10.3390/jcm11092435)
Supplement: Supplementary file 1 [file jcm-11-02435-s001.zip › Supplementary File S2/HDTL_Affiche_Inhibiteurs Pompe_Protons_A4.pdf]

Hôpital de La Tour

Revoir la poursuite d'un traitement à long terme d'inhibiteurs de la pompe à protons

Le saviez-vous ?

Les inhibiteurs de la pompe à protons (IPP) comptent parmi les médicaments les plus souvent prescrits

50%

Dans plus de 50% des cas, cette prescription est inappropriée

Les inhibiteurs de la pompe à protons sont associés à des effets secondaires non négligeables

L'impact sur les coûts de la santé est important

Effets secondaires reconnus

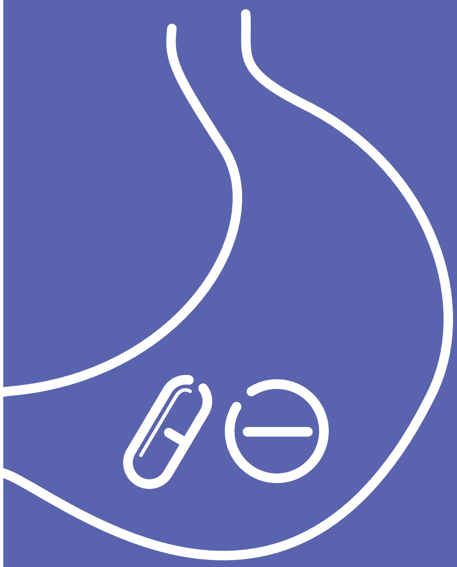

- Fracture des os
- Effet rebond à l'arrêt du traitement
- Maladies rénales chroniques
- Pneumonies
- Diarrhées infectieuses
- Carences en Calcium, Magnésium, vitamine B12 et en Fer

Mauvaises indications

» Traitement prolongé de reflux sans essai de sevrage

» Prévention ulcère chez patient sous cortisone ou sous AINS sans facteur de risque

» Prévention ulcère de stress à l'étage
