# Supplementary material for: Implementing a Multifaceted Intervention among Internal Medicine Residents with Audit and Educative Data Feedback Significantly Reduces Low-Value Care in Hospitalized Patients
Source: J Clin Med. 2022 Apr 26;11(9):2435. doi: 10.3390/jcm11092435 (PMC9104072; doi:10.3390/jcm11092435)
Supplement: Supplementary file 1 [file jcm-11-02435-s001.zip › Supplementary File S2/HDTL_Affiche_testsanguin_A4.pdf]

Hôpital de La Tour

Réduire le nombre d'examens de laboratoire

Le saviez-vous ?

De nombreux examens de laboratoire sont demandés à intervalles réguliers (parfois quotidiennement)

Un examen de laboratoire devrait être demandé uniquement pour répondre à une question clinique précise

La prescription régulière des tests augmente les coûts de la santé, ne profite pas aux patients et peut leur nuire

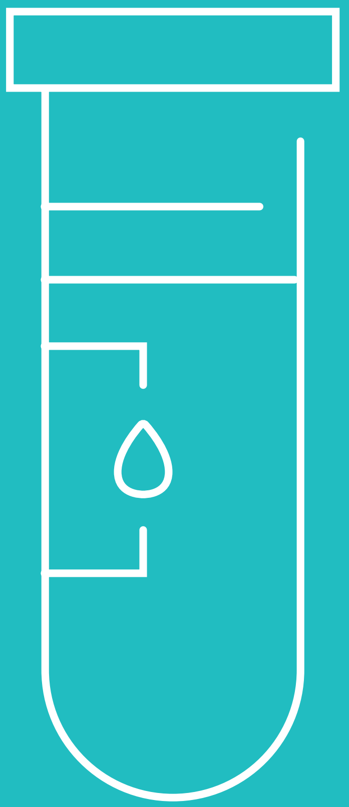

Complications

- Coûts
- Anémie
- Prolongation du séjour hospitalier
- Examens complémentaires secondaires inutiles
