# Supplementary figures and images for: Implementing a Multifaceted Intervention among Internal Medicine Residents with Audit and Educative Data Feedback Significantly Reduces Low-Value Care in Hospitalized Patients
Source: J Clin Med. 2022 Apr 26;11(9):2435. doi: 10.3390/jcm11092435 (PMC9104072; doi:10.3390/jcm11092435)

Prescription rates of proton pump inhibitors treatment

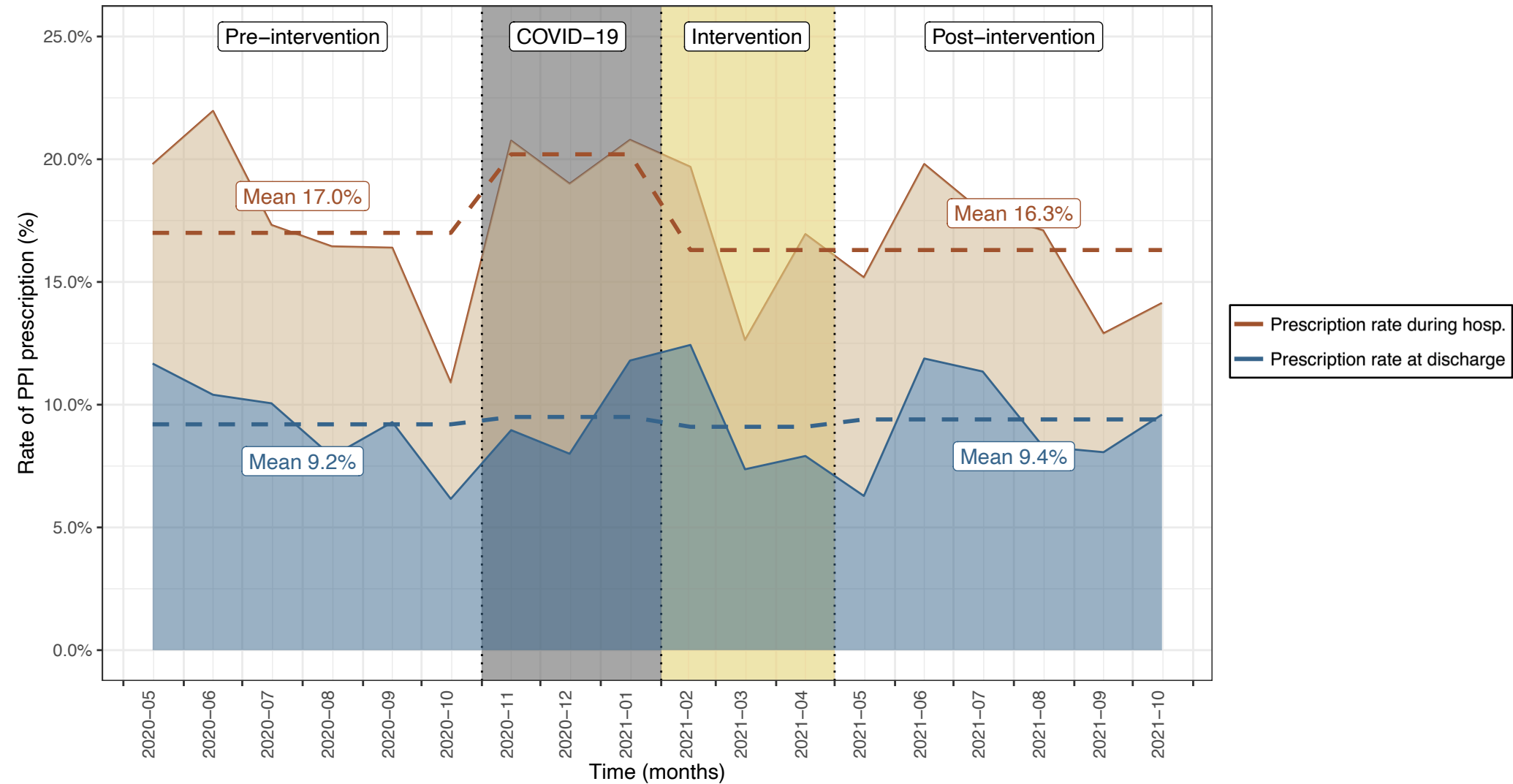

Supplement: Supplementary file 1 [file jcm-11-02435-s001.zip › Supplementary File S3.pdf]
